# Uncovering Polyoxometalate Speciation in Hydrothermal Systems by Combining Computational Simulation with X‑ray Total Scattering

**DOI:** 10.1021/jacs.5c04696

**Published:** 2025-06-17

**Authors:** Laura S. Junkers, Diego Garay-Ruiz, Jordi Buils, Rebecca S. Silberg, Guilherme B. Strapasson, Kirsten M. Ø. Jensen, Carles Bo

**Affiliations:** † Department of Chemistry, 4321 University of Copenhagen, Universitetsparken 5, Copenhagen 2100, Denmark; ‡ Institute of Chemical Research of Catalonia (ICIQ), 202569 Avinguda Països Catalans 16, Tarragona 43007, Spain; § Departament de Química Física i Inorgànica, Universitat Rovira I Virgili (URV), Marcel·lí Domingo, 1, Tarragona 43007, Spain; ∥ Institute of Chemistry, University of Campinas, Rua Monteiro Lobato 270, Campinas SP 13083-862, Brazil; ⊥ Brazilian Synchrotron Light Laboratory, CNPEM, R. Giuseppe Máximo Scolfaro, 10000, Campinas SP 13083-100, Brazil

## Abstract

A systematic approach for understanding the pH-dependent speciation of molecular metal-oxide nanoclusters beyond ambient conditions, which combines computational predictions with X-ray total scattering experiments, is presented. We demonstrate that temperature-dependent water properties have a significant impact on molecular energies derived from implicit solvent modeling and propose an efficient correction strategy. Based on this, we expand our methodology toward the elevated temperatures and pressures characteristic of hydrothermal synthesis. Correlating these computational results with experimental observations reveals a remarkable synergy between the two approaches, which helps to differentiate closely related polyoxometalates coexisting in solution. We find that qualitative trends are directly reproduced computationally, while the intricate nature of polyoxometalate speciation is best captured by adjusting computational predictions based on experimental insights. The derived knowledge of the clusters present under various conditions enables us to rationalize the crystallization of h-MoO_3_ at high temperatures and very acidic pH. With this, our study highlights the potential of hybrid approaches for elucidating solution-based oxide formation under extreme conditions.

## Graphical abstract

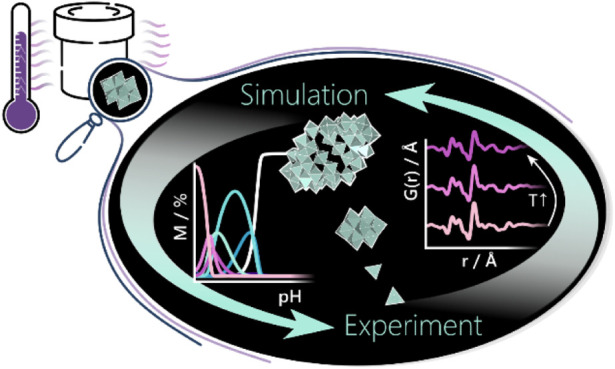

## Introduction

Tailoring hydrothermal synthesis requires knowledge about the species and chemical processes involved. For metal oxides formation, these species frequently include discrete molecular metal-oxo clusters. Specifically, aqueous solutions of fully oxidized group V and VI transition metals, like V^V^, Mo^VI^ or W^VI^, give rise to discrete metal-oxo clusters known as polyoxometalates (POMs).^1^ This umbrella term covers a multitude of clusters, varying in size, shape, and chemical composition. Accordingly, POM chemistry targets a wide field of applications spanning from protein crystallography^2^ and medicine^3–5^ over catalysis^6–10^ to energy storage.^11^ Moreover, magnetic POMs have been explored in the context of quantum computing.^12–14^ The self-assembly of POMs from solution relies on vast reaction networks^15^ which are sensitive to ionic strength, temperature, redox agents, and counterions.^16–18^ Particularly pH and metal concentration lend themselves for controlling POM speciation, which typically involves multiple coexisting clusters.^1^ The complexity of aqueous POM chemistry makes it challenging to predict and rationalize the reaction pathways involved in POM speciation.^15^ Therefore, understanding their aqueous speciation under synthesis conditions is essential for enhanced synthesis control.

We recently introduced a new computational method named POMSimulator^15,19^ which enables us to handle the complex reaction networks underlying aqueous POM speciation, and predict speciation diagrams under ambient conditions. As visualized in Figure 1a, the initial version of POMSimulator relies on both experimental and computational input. Density Functional Theory (DFT) provides optimized molecular geometries, electron densities, and the corresponding Gibbs free energies. Experimental formation constants, K_f_, are then used for rescaling the computationally predicted K_f_ values. Beyond this version, statistical methods^20^ and the exploration of universal scaling trends across various systems^21^ have enabled advancements toward decoupling POMSimulator from experimental data. Our method constructs the chemical reaction networks representing the plethora of equilibria involved in POM speciation, and subsequently builds and solves speciation models. This makes it possible to predict the abundance of specific clusters under a continuum of conditions (Figure 1b). The methodology has been shown to reliably predict pH- and concentration-dependent speciation for isopolyoxometalates of Mo, W, V, Nb, and Ta,^22,23^ and for heteropolyoxometalates (PMo)^20^ and (AsMo),^21^ based on the aforementioned statistical treatments.

**Figure 1:**
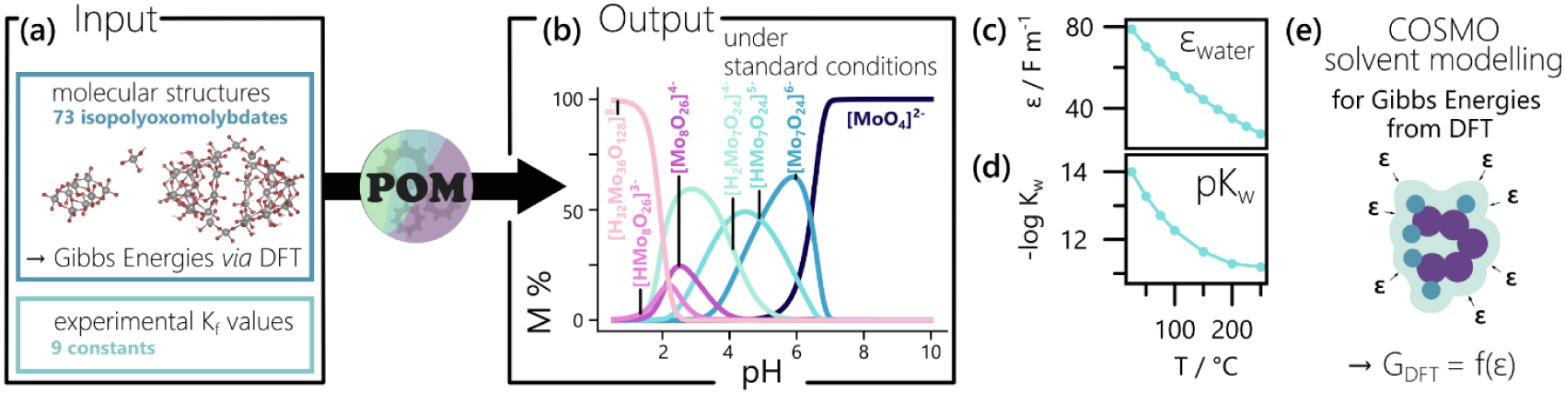
(a) Simplified overview over the input needed for predicting isopolyoxomolybdate speciation with POMSimulator. (b) Output of POMSimulator, exemplarily visualized in form of the predicted speciation diagram of an aqueous Mo^VI^ solution with [Mo] = 1 M under standard conditions. For more detailed schemes on the POMSimulator workflow, the reader is referred to our previous publications.^15,19^ (c) and (d) show the influence of increasing temperature on (c) the dielectric constant ε,^24^ assuming a pressure of 100 bar, and (d) the autoprotolysis constant of water, p*K*_w_, assuming saturated vapor pressure.^25^ (e) Schematic representation of the role the dielectric constant of the solvent plays in the calculation of molecular energies of dissolved clusters using a dielectric continuum model like COSMO.^26^ A more complete visualization of the COSMO approach is shown in Figure S2.

Up until now, POMSimulator has only been applied to aqueous solutions under ambient conditions. However, the properties of water change rapidly with respect to temperature and, to a lesser degree, pressure.^27^ This effect is rooted in the interaction between water molecules,^28^ since an increase in temperature causes a drop in the number of hydrogen bonds per water molecule.^29^ This change is reflected in the static dielectric constant, ε, of water which drops rapidly upon heating, as Figure 1c shows.^24^ When approaching temperatures of 150 – 250 °C, water thereby gradually adopts polarities akin to organic solvents like acetonitrile or dimethylformamide.^27^ Simultaneously, its autoprotolysis constant (p*K*_w_) decreases, as shown in Figure 1d.^25^ Heating also entails a decrease in viscosity^30^ and density,^31^ shown in Figure S1a, which primarily impacts diffusion-controlled reactions.^27^

Hydrothermal synthesis capitalizes on this impact of temperature and pressure on water.^27,32^ Commonly starting from simple metal salt solutions, heat and pressure, i.e., hydrothermal conditions, are applied to induce particle nucleation. Rooted in geochemistry, the hydrothermal method is a particularly successful route for the tunable synthesis of oxide nanoparticles,^33^ including the oxides of metals which are prone to POM formation.^34–37^ The involvement of POMs and POM-related cluster species in the hydrothermal formation pathways has been extensively demonstrated for tungsten oxide-based materials.^38–41^ Moreover, cluster speciation right before tungsten oxide crystallization was found to facilitate polymorph control.^40^ These findings showcase the relevance of studying POM speciation beyond ambient conditions.

Common experimental techniques for determining POM speciation in solution include nuclear magnetic resonance (NMR),^42–44^ potentiometric titrations,^45,46^ mass spectrometry,^47^ and vibrational spectroscopy.^48,49^ Moreover, the size and shape distribution of clusters as well as their interactions can be studied via small-angle X-ray scattering (SAXS).^50^ Particularly Nyman and coworkers widely applied SAXS to reveal valuable insights into POM speciation.^51–53^ In studying small POMs with diameters of around 1 nm, the lower size limit of SAXS has repeatedly been approached.^50^ This limitation can be overcome through X-ray total scattering (TS), which has been demonstrated as an efficient probe for POM structures, both in combination with SAXS^54,55^ and in stand-alone TS studies.^38,40^ While SAXS probes cluster sizes in the nm range, X-ray total scattering combined with pair distribution function (PDF) analysis enables the assessment of local atomic structures, from distances between neighboring atoms (<3 Å) up to insight into interatomic distances across a few nm.^56^ Compared to conventional crystallography methods, PDF analysis allows the study of structures regardless of crystallinity, which makes *in situ* X-ray total scattering particularly interesting for following material formation.^57^ Accordingly, this experimental method plays a vital role in investigating POM speciation during hydrothermal oxide formation.^38–40^ However, these experiments entail advanced data analysis, which hinges on identifying suitable structural models.^58^

This bottleneck of experimental data analysis can be addressed by supporting the analysis with computational methods. For example, the AI/ML classifier POMFinder can accelerate experimental data analysis by screening crystallographic databases for POM-like motifs that match an experimental PDF.^58^ However, this approach solely relies on structural characteristics and thereby neglects essential chemical parameters such as pH in POM identification. This impedes the differentiation of multiple coexisting POMs from one novel cluster species. Chemically informed methodologies like POMSimulator address exactly these limitations and therefore hold immense potential for complementing experimental studies with additional insight into the intricate equilibria of POM speciation.

Here, we expand the established POMSimulator methodology to unravel the speciation of isopolyoxomolybdates beyond ambient conditions. Our results emphasize the added value derived from combining structural insights from X-ray total scattering experiments with chemically informed computational tools.

## Methods

### Computational Details

Our computational work relies on a previously reported set^22^ of isopolyoxomolybdate structures (73 species) referred to as “Mo set” in the following, and the same subset of experimentally reported species as used by Petrus *et* Bo for speciation and speciation phase diagram simulation.^22^ The complete data set^59^ is accessible in the ioChem-BD repository,^60^ as is all new data generated in this work.^61^

For the geometries retrieved from the data set, we carried out unconstrained geometry optimizations and frequency calculations using the ADF 2019 software^62^ employing the Perdew–Burke–Ernzerhof (PBE) functional^63,64^ and a TZP basis set, including the Zero-Order Regular Approximation (ZORA)^65,66^ for relativistic effects as well as the Conductor-like Screening Model (COSMO)^26^ for solvation using the atomic radii from Klamt.^67,68^ The frozen core was set to Large for the optimization and frequency calculations, in consistency with previous results.^22^ Single-point energy calculations with varying solvation parameters and a Small frozen core were employed to model high temperature effects. Experimental formation constants (K_f_) were adjusted for temperature via eq 1, assuming a temperature-independent reaction free energy, ΔG_r_. Reference values from Cruywagen were used (Table S1).

1 $$\log K_{f}\left(T\right)=-RT\Delta G_{r}$$

We employed the open-source version of POMSimulator, incorporating the temperature as a variable parameter.^19,69^ Simulated pH values were restricted to a range of 0 – 10, strictly avoiding limit regions (pH > 12) where the pH would exceed the p*K*_w_ value of water at the highest temperature in the study (125 °C). The ionic strength was set to 1.0 M.

### Experimental Methods

Aqueous solutions of Na_2_MoO_4_ · 2 H_2_O were prepared with a concentration of [Mo] = 1 M and pH values of 1.6, 3.4, or 5.6. While ensuring Mo^VI^ concentration control, the pH was adjusted with 6 M HCl and determined using a HANNA HI 2020–02 pH meter. Hydrochloric acid (37%) and Na_2_MoO_4_ · 2 H_2_O (purity: ≤ 99%) were purchased from VWR chemicals and Sigma-Aldrich, respectively, and used as received. All solutions were prepared within 2 h prior to the measurements.

### Time Resolved X-ray Total Scattering Experiments

The X-ray total scattering (TS) experiments were conducted at the DanMAX beamline at MAXIV in Sweden using a wavelength of 0.3542 Å. We used the rapid acquisition PDF (RA-PDF) method,^70^ where a large 2D area detector is placed closely (∼ 16.5 cm) behind the sample, to collect TS data *in situ* with a time resolution of 10 s. We used a custom capillary reactor described by Roelsgaard et al.:^71^ A thin silica capillary (diameter: 0.7 mm, wall thickness: 0.09 mm) was fixed in a custom steel frame and linked to steel tubing via Swagelok fittings. After injecting the sample solutions into the capillary, we applied 100 bar of pressure using a commercial high-performance liquid chromatography (HPLC) pump. With a hot-air blower located below the capillary, the temperature was stepwise increased from ambient temperature, to 75 °C, and 125 °C. To ensure that equilibria were probed, we measured for 3 min at ambient temperature and 10 min each at 75 °C and 125 °C, as schematically depicted in Figure S3. Data acquisition was paused while transitioning between temperatures with 100 °C/min.

### Data Corrections and Integration

The 2D TS data frames were calibrated and integrated using pyFAI.^72^ We subtracted scattering patterns of pure water in the setup under the same conditions (p and T) as our sample solutions as background. This subtraction as well as the data correction, normalization, and Fourier transform involved in making PDFs were done using PDFgetX3.^73^ The PDFs were obtained with r_poly_ = 0.9, *Q*_max_ = 14 Å^–1^, *Q*_maxinst_ = 16.8 Å^1^, and a nominal composition of Na_2_MoO_4_. To minimize small-angle scattering contributions, evident in Figure S4, *Q*_min_ was set to 1.5 Å^–1^. Instrumental parameters were determined using a Si standard (*Q*_damp_ = 0.0228 Å^–1^, *Q*_broad_ = 0.0012 Å^–1^).

### Data Analysis

The DFT-derived molecular structures^22^ used in POMSimulator function as structural models for our PDF analysis. Whenever possible, cutouts from previously reported crystal structures were used for comparison, as detailed in the Supporting Information. Simulated PDFs were calculated using DebyeCalculator^74^ and all cluster refinements performed using Diffpy-CMI.^75^ Only the overall scale factor was refined. Refinements of the crystalline phase were done in PDFgui.^76^ We used Pearson correlation matrices to visualize and quantify the similarity between all combinations of frames in each PDF data set.^77^ All molecular structures were visualized using the VESTA software.^78^

## Results and Discussion

The first step of the study was the expansion of POMSimulator beyond ambient conditions. To this end, we discuss the influence of temperature-induced solvent changes on molecular Gibbs free energies, before moving on to the resulting speciation predictions for isopolyoxomolybdates. Subsequent analysis of X-ray total scattering data provided deeper insight into the same system and served to assess the predictive power of POMSimulator under hydrothermal conditions.

### Computational Predictions

For exploring hydrothermal conditions, we considered a temperature range from 25 to 125 °C at a high pressure of 100 bar, as used in the *in situ* TS setup. Within the covered temperature range and at the target pressure, the dielectric constant of water (ε) almost halves, as shown in Figure 1c. Simultaneously, changes in density (ρ) occur, which impact the solvent radius $\left(R_{solv}\right)$ , as shown in Figure S1. Both ε and $R_{solv}$ are the key parameters in the implicit solvent model approach COSMO,^26^ applied in this work (Figure 1e and S2). Thus, the Density Functional Theory (DFT) calculations needed for POMSimulator can be strongly affected by temperature-dependent solvent properties. The assessment and consideration of their impact is, therefore, crucial for the reliable prediction of POM speciation at high temperatures.

The strategy schematized in Figure 2 efficiently accounts for temperature-induced solvent changes: single-point energy calculations at the stationary points are carried out with *T*- and P-corrected ε and $R_{solv}$ parameters to obtain reliable electronic energies, $E_{el}^{*}$ . To limit the computational cost, free energy correction (G_corr_) terms are calculated from the original (non-T,P-corrected) frequency calculations. The proposed hybrid approach was shown to be viable for a test set containing several isopolyoxomolybdates (see Section 1 of the Supporting Information). Moreover, we found that accounting for the temperature dependence of COSMO parameters significantly affects the formation energies of the probed POMs (further discussed in Section 2 of the Supporting Information). Since neither the temperature nor molecular characteristics such as charge or size can be directly correlated with the observed changes, they cannot be addressed via simple extrapolation approaches. In summary, the proposed strategy (Figure 2) is a reasonable approximation for the correction of DFT-based energies in aqueous solutions at high temperatures and pressures.

**Figure 2:**
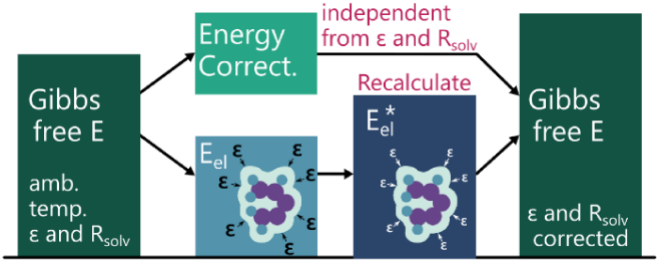
Schematic representation of the calculation strategy used throughout this study, which allows to efficiently consider temperature- and pressure-dependent water properties in the calculation of molecular Gibbs free energies. $E_{el}$ is the electronic energy at ambient temperature and pressure, and $E_{el}^{*}$ , the electronic energy computed with water COSMO parameters at the target (T,P).

Using this strategy, we calculated the Gibbs free energies of the “Mo set” (listed in Table S2), as well as water, the hydronium ion and the Zundel pair (H_5_O_2_^+^/H_4_O_2_), across three different temperatures (T = 25 °C, 75 °C, and 125 °C) and applied POMSimulator to predict the respective formation constants. This enables us to consider not only the changes in the stability of the molybdates, but also the changes in the solvation of the proton. At this point, the concept of speciation models comes into play. In the context of POMSimulator, these models correspond to reaction subsets containing the same number of species and equations (in mathematical terms, a determined system of equations), so that each speciation model represents a mechanistic proposal, with at least one reaction leading to each cluster in the data set. A plethora of speciation models contributes to POMSimulator results (here: N_models_ = 466k), each yielding one formation constant for each molecular species (N_species_ = 73). Accordingly, each set of predicted constants has dimensions of N_species_ x N_models_.

To navigate the wide variety of generated models, two main approaches have been followed to date. The first one selects the “best” model based on a direct fit to experimental reference values, assessed through the Root Mean Squared Error (RMSE).^15^ Alternatively, statistical pipelines have been applied to group similar models and average them.^20^ In this work, we followed an intermediate approach: we preserved the RMSE-based selection of models, aiming to easily compare our results with the previously reported speciation of molybdates under ambient conditions,^22^ but included a certain degree of variability by selecting the best **n** models and averaging their speciation. We determined that **n** = 25 provided a good balance between the number of speciation diagrams to solve, which increases the overall cost, and the robustness of the results, as detailed in Section 3 of the Supporting Information.

Applying the chosen averaging approach gives rise to speciation phase diagrams (Figure 3), which show only the most abundant cluster structure adopted by Mo^VI^ at each combination of pH and total Mo^VI^ concentration. When comparing Figure 3a–c, it becomes apparent that the pH/concentration range at which polynuclear POM species occur shrinks with increasing temperature. For example, a temperature increase from 25 to 125 °C shifts the lowest concentration at which POMSimulator predicts abundance of diprotonated {Mo_7_} (“**6**”) from [Mo] = 10^–3.5^ M up to [Mo] = 10^–2.5^ M, as evidenced by Figure 3a and c.

**Figure 3:**
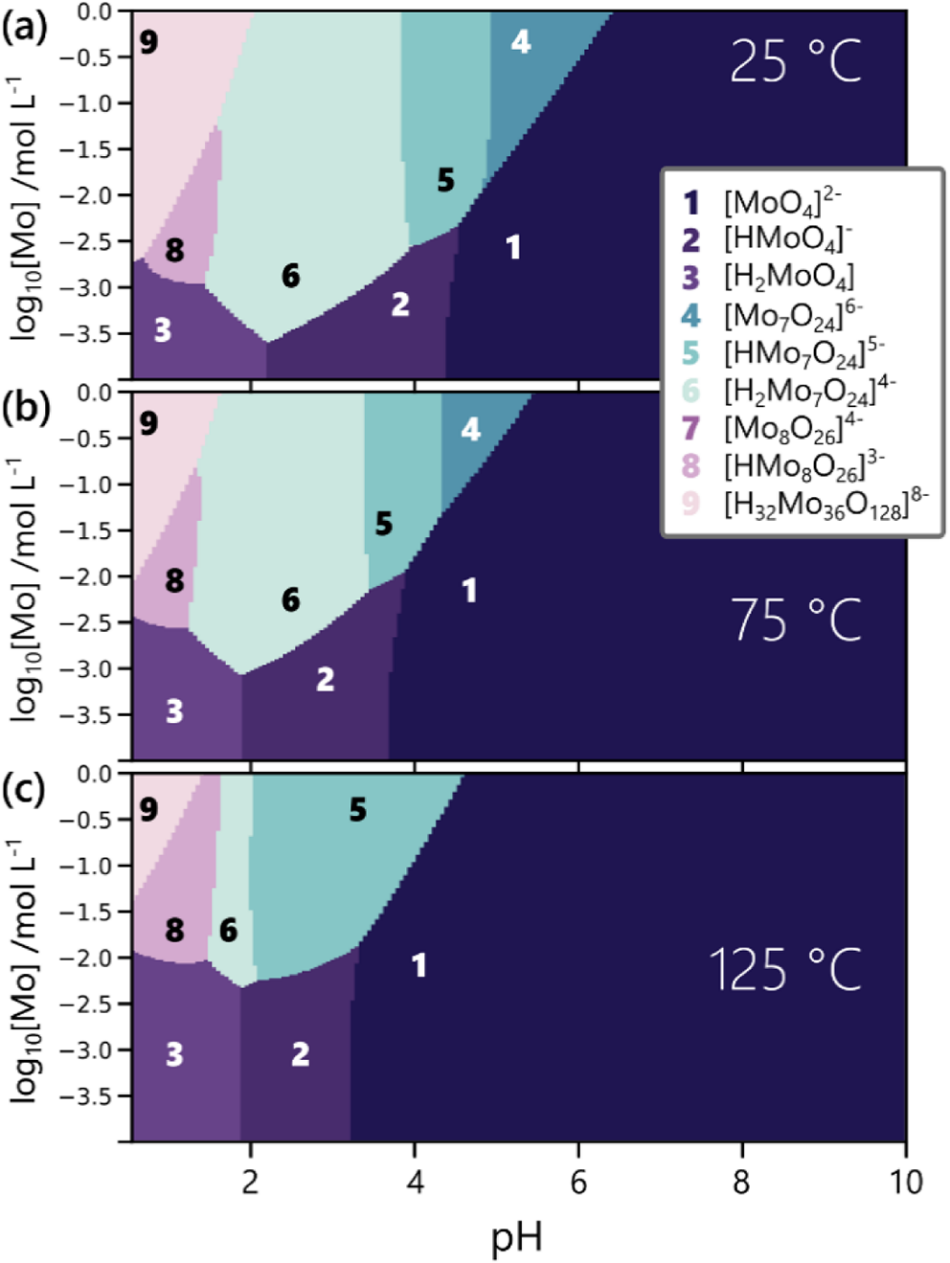
Comparison of the speciation phase diagrams obtained by averaging over the 25 best models at each temperature individually. The probed temperatures include (a) 25 °C, (b) 75 °C, (c) 125 °C. Each speciation phase diagram is a stacked representation of numerous speciation diagrams like the one depicted in Figure 1b, varying in [Mo] which is logarithmically plotted along the *y*-axis. The most abundant cluster species, i.e., the one binding the largest share of Mo^VI^ at a given pH/[Mo] combination, is encoded using both a color code and numbers.

Moreover, increasing the temperature limits POM formation to lower pH values, as evident from the stability regions of {Mo_7_}. For 25 °C, unprotonated {Mo_7_} (“**4**”) is predicted as prevalent until beyond pH = 6 (Figure 3a), while {Mo_7_}, this time protonated (“**5**”), is only predicted below pH = 5 at 125 °C (Figure 3c).

Our predictions therefore imply that increasing temperature stabilizes the monomer [MoO_4_]^2–^ and its protonated forms relative to clusters of higher nuclearity. Furthermore, the phase diagrams in Figure 3 indicate that the formation of {Mo_36_} at high temperatures requires increasingly high [Mo] and low pH values.

### Predicted Speciation at Experimental Conditions

Linking the discussed phase diagrams with experiments requires us to focus on one specific Mo^VI^ concentration (i.e., one horizontal row in a phase diagram) matching the experiments, i.e., [Mo] = 1 M (log_10_[Mo] = 0.0).

Thus, the respective speciation diagrams (Figure 4) correspond to the topmost row of the phase diagrams in Figure 3. All predicted species are now depicted, providing a different representation of the temperature-dependent trends seen in Figure 3. Large cluster species get destabilized upon heating, as exemplified by the onset of {Mo_36_} formation shifting from pH = 2.5 at 25 °C (Figure 4a) to pH ∼ 1.5 at 125 °C (Figure 4c). Simultaneously, the [MoO_4_]^2–^ monomer becomes dominant over a wider pH range, with its lower limit expanding from pH = 6.5 down to pH = 5. This behavior might be related to the reduced polarity of water under hydrothermal conditions as well as the increased importance of entropic effects at elevated temperatures, since both effects disfavor the presence of highly charged, large POM species. More ambiguous changes are seen in the distribution of {Mo_7_} and β-{Mo_8_} species with varying degrees of protonation, which suggests that their close resemblance in charge and nuclearity hinders the reliable prediction of individual stabilization trends.

**Figure 4:**
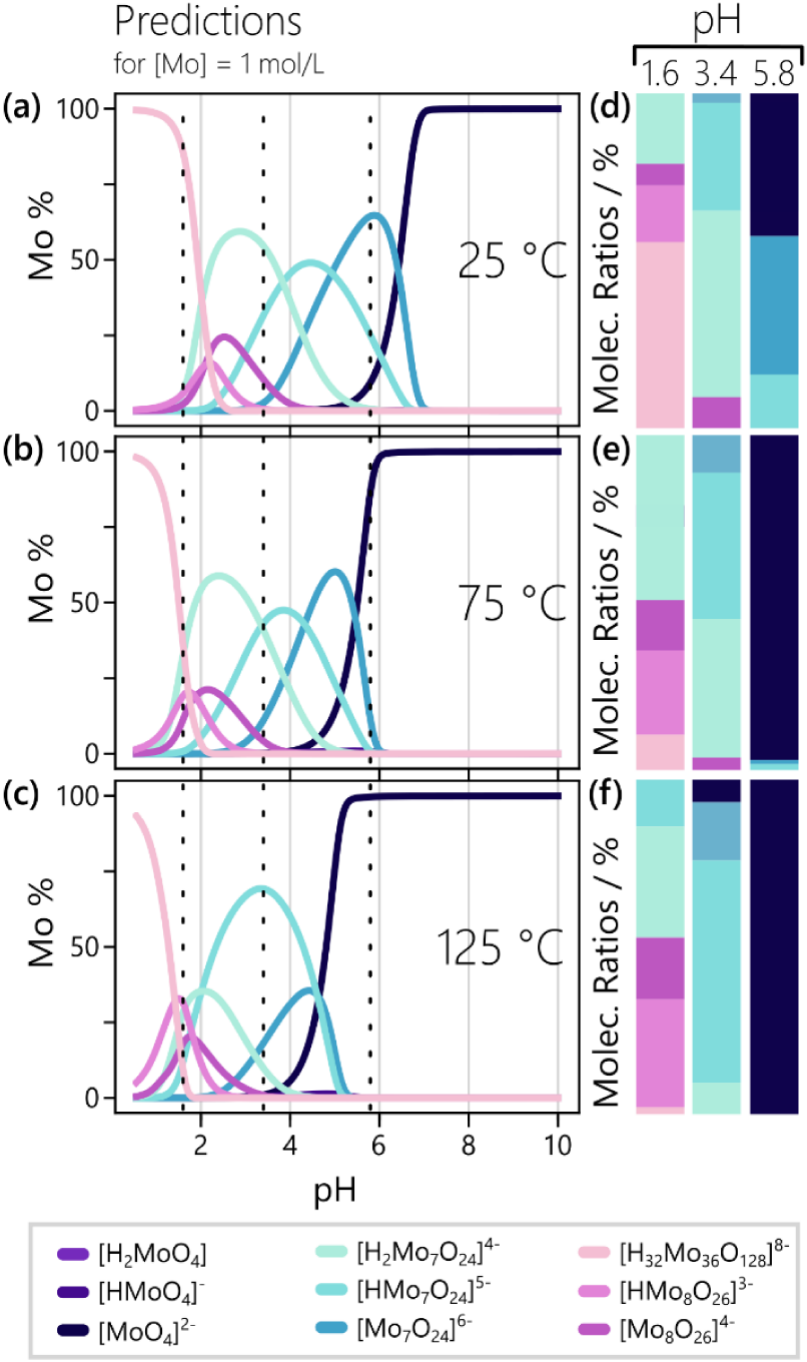
Predicted POM speciation in a 1 M aqueous Mo^VI^ solution at various temperatures. These results are based on averaging the 25 best speciation models. The shown speciation diagrams for (a) 25 °C, (b) 75 °C, and (c) 125 °C can be understood as another representation of the topmost horizontal line of the corresponding phase diagrams, shown in the respective subplots of Figure 3. Vertical dotted lines highlight the pH values assessed experimentally. The corresponding molecular ratios of all Mo^VI^ species, used as scale factors in the experimental data analysis, are shown for (d) 25 °C, (e) 75 °C, and (f) 125 °C and listed in Table S4.

To enable the experimental validation of POMSimulator through direct comparison between predictions and experimental results, we extracted the molecular ratios from each speciation diagram in Figure 4 (details in Section 4 of the Supporting Information). The resulting ratios, which function as scale factors in the subsequent PDF analysis, are visualized in Figure 4d-f and listed in Table S4.

### Experimental Results

We collected time-resolved X-ray total scattering of aqueous Na_2_MoO_4_ solutions with a concentration of [Mo] = 1 M using a total of nine experimental conditions. To cover a wide pH range, we probed pH values of 1.6, 3.4, and 5.8. Our experiments include three different temperatures, namely ambient temperature (amb. temp.), 75 °C, and 125 °C. All PDFs obtained for one temperature/pH combination make up a data set. The thermodynamics-based equilibria described by POMSimulator can only be compared to experiments probing equilibria. We confirm this by comparing the first and last frame of each data set, which cover 3 min for ambient temperature data and 10 min for elevated temperatures.

The limited time span of our measurements does not allow us to rule out slowly establishing equilibria. However, previous work by Cruywagen et al. emphasizes the fast nature of isopolyoxomolybdate self-assembly,^79^ which makes it plausible that equilibria are probed. For all but one data set, our data indicates that equilibria were already established when the first PDF frame was collected, as evidenced by the negligible features of the gray difference curves in Figure 5a-c. Throughout our measurement, only the pH = 1.6 data collected at 125 °C changes and the long correlation length of the “Last” PDF for 125 °C in Figure 5a indicates heat-induced crystallization. The formed structure was identified as h-MoO_3_ ^80^ (visualized in Figure S10a), as evidenced by Figure 5d, and remnants of the initial POM speciation are present up until the end of the experiment (3 min at 125 °C). Details on the h-MoO_3_ crystallization are discussed in Section 5 of the Supporting Information.

**Figure 5:**
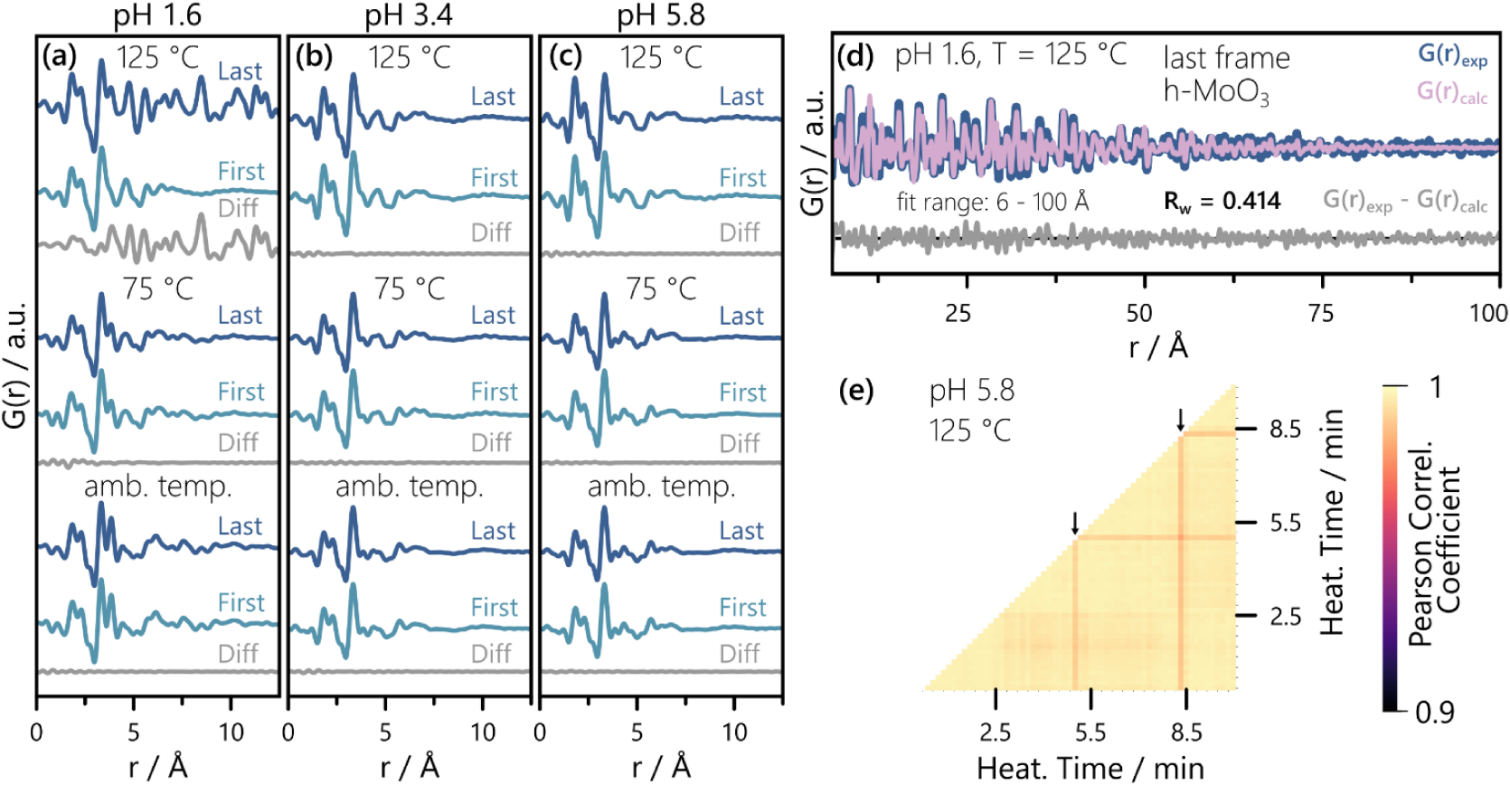
First and last PDF frames of each data set as well as their difference for (a) pH = 1.6, (b) pH = 3.4, and (c) pH = 5.8. (d) PDF refinement of h-MoO_3_ ^80^ to the final PDF for pH = 1.6 and T = 125 °C (fit range: 6 to 100 Å). Scale factor, lattice parameters, crystallite size, and a common isotropic displacement parameter of the Mo atoms were refined. Fit parameter results are listed in Table S5. (e) Pearson correlation matrix of the pH = 5.8, T = 125 °C data, where dark purple and yellow stand for low and high Pearson Correlation coefficients, respectively. Two identical PDFs yield a coefficient of 1 and complete lack of similarity yields a coefficient of 0. Analogous plots for the remaining eight data sets are given in Figures S11–S13. The small vertical arrows in (e) highlight frames that differ from their neighbors, which are further assessed in Figure S15.

Along with the PDF comparison in Figure 5a-c, we used Pearson Correlation matrices to quantify the similarity between all possible pairs of PDFs within each data set. This representation of our PDF data confirms that equilibria are established under all sets of conditions except for pH = 1.6 at 125 °C, as evidenced by the correlation matrices of all nine data sets collected in Figures S11–S13. Details on the matrix covering the crystallization of h-MoO_3_ (pH = 1.6, 125 °C) are discussed in Section 5 of the Supporting Information. All other Pearson Correlation matrices exhibit a lack of clear trends and overall light colors, representing Pearson Correlation coefficients close to 1, which stands for complete agreement between two functions. For reference, a complete lack of similarity would yield a coefficient of 0, yet the color code in Figures S11 - S13 only spans from 0.9 to 1. While the overall absence of significant changes agrees with the gray difference curves in Figure 5a-c, individual frames differ noticeably from their neighbors, as highlighted exemplarily for the pH = 5.8 at 125 °C data in Figure 5e. These variations are linked to an increased noise level in selected frames, as evidenced by the PDF comparison in Figure S15, and can be attributed to beam fluctuation.

In summary, high temperatures combined with a low pH induced h-MoO_3_ ^80^ crystallization. Therefore, our data indicates that equilibria were probed across all conditions except pH = 1.6 at 125 °C, causing this data set to be excluded from comparison with computational results. For improved signal-to-noise ratio, we summed the remaining eight data sets over their complete measurement time, i.e., over 3 or 10 min, respectively.

With experimental data suitable for comparison with computational predictions, the next step is to test our structural models. Specifically, we investigated how reliable the DFT-derived POM geometries are under the lens of X-ray PDF compared to POM structures determined from single crystal crystallography. Such structures are available for unprotonated {Mo_7_}, β-{Mo_8_}, and {Mo_36_}. When comparing simulated PDFs of the two sets of structures, we see significant differences for the β-{Mo_8_}, and {Mo_36_} pairs, especially around the metal–metal peaks at ∼ 3.3 Å and ∼ 3.8 Å, as evidenced by Figure 6a. Nonetheless, equivalent structures closely resemble each other, as evidenced by the visual comparison in Figure S5c–f. The minor mismatches observed in their PDFs may arise either from the geometry optimizations ending in local minima of the complex energy landscape, or from slight differences between POM structures in solution and in the solid state.

**Figure 6:**
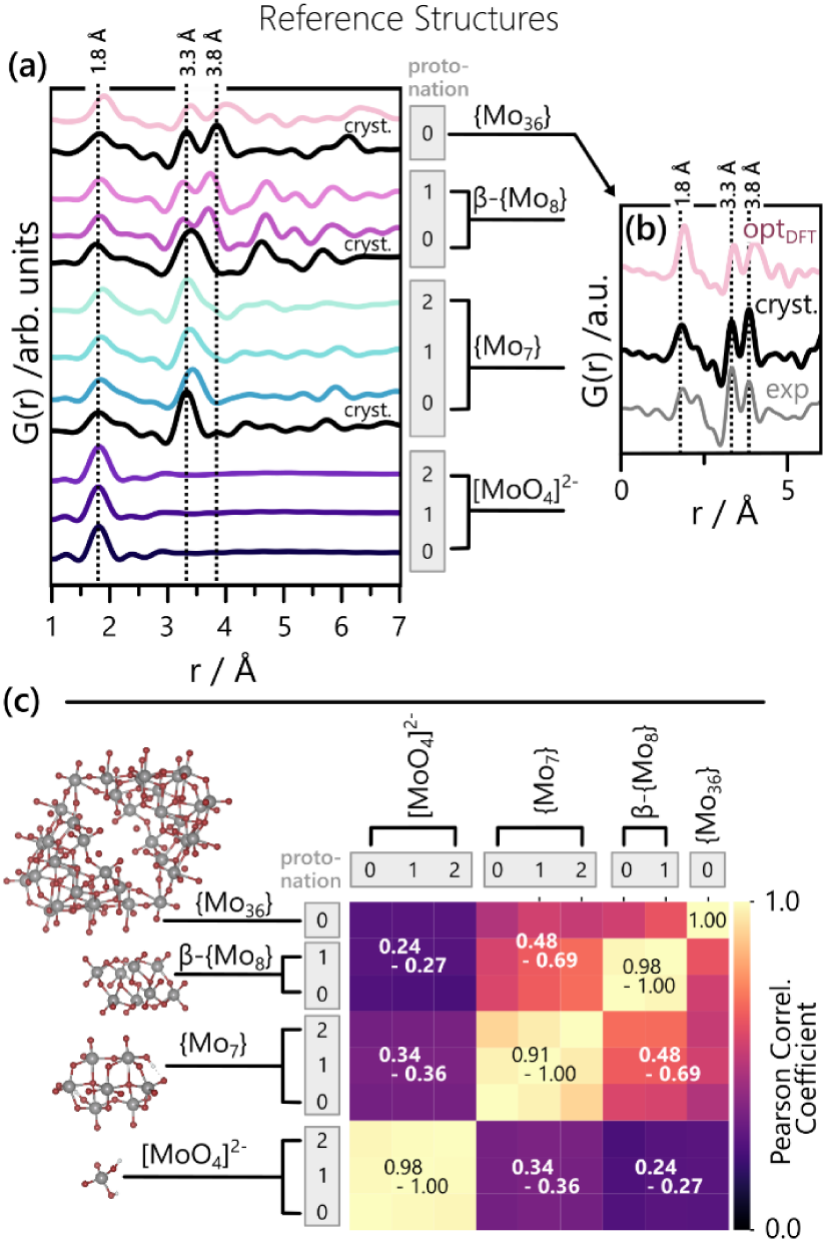
(a) Overview of the reference PDFs used for the experimental data analysis, compared side-by-side, using the color code introduced in Figure 4 for PDFs of all cluster structures included in the speciation models. Additionally, black reference PDFs, marked as “cryst.” are included, representing the unprotonated structures of {Mo_7_}, β-{Mo_8_}, and {Mo_36_} in solid-state (structures detailed in the Supporting Information). This enables direct comparison between solid-state and DFT-derived POM PDFs. Dotted vertical lines highlight the characteristic distances for Mo–O (1.8 Å) and Mo–Mo (3.3 Å and 3.8 Å) pairs. The shown plot covers an r-range from 1 to 7 Å. Figure S16a compares the same data over a wider r-range. (b) In-depth comparison between the solid-state and DFT-derived {Mo_36_} cluster via their PDFs. The experimental PDF of the pH = 1.6 sample at ambient temperature (summed over 3 min) is given for comparison. (c) Pearson correlation matrix, quantifying and visualizing the similarity between all possible pairs of the reference PDFs, shown in more detail in Figure S16b. The solid-state cutout of {Mo_36_} was used here.

For {Mo_36_}, we can directly determine which of the two structural models describes the experimental {Mo_36_} PDF best, since the cluster is known to dominate solutions with low pH values (pH ∼ 1)^1^ under ambient conditions. Particularly at ∼ 3.8 Å, the solid-state structure of {Mo_36_} describes the PDF observed for pH = 1.6 at ambient temperature better, as evidenced by Figure 6b. This confirms that the DFT-optimized geometry of {Mo_36_} corresponds to a local energy minimum, caused by size and complexity of the cluster. Consequently, we used the solid-state {Mo_36_} structure for the PDF analysis. However, POMSimulator adequately predicted the overall solution behavior at ambient temperature (dominance of {Mo_36_} at low pH and high concentrations), indicating that the {Mo_36_} geometry mismatch did not significantly impact the respective DFT energy. Therefore, we refrained from changing the {Mo_36_} structure used for POMSimulator. Aside from {Mo_36_}, DFT-derived geometries were used for the PDF analysis.

For this set of POM structures, we now assess which of them can be structurally differentiated via X-ray PDFs. Some POMs can be easily distinguished, as evidenced by Figure 6a where all nine predicted species are compared based on their PDFs. Initial visual comparison already reveals that {Mo_36_} clearly differs from {Mo_7_} and [MoO_4_]^2–^. On the other hand, clusters of the same nuclearity, such as {Mo_7_} clusters with different degrees of protonation, give rise to highly similar PDFs. This is in line with the intrinsically low scattering strength of protons but also highlights that the impact of protonation on the Mo positions of isopolyoxomolybdates is small.

These qualitative observations are underpinned by the Pearson Correlation matrix in Figure 6c, which confirms that we cannot differentiate between protonation states (yellow blocks on diagonal) but should be able to distinguish PDFs of POM differing in their nuclearity (darker off-diagonal blocks).

However, the mathematical comparison of two simulated PDFs is only a simplified measure of distinguishability within experimentally probed POM speciation, which typically involves multiple coexisting clusters. In experimental data, the nine predicted clusters are therefore primarily identified based on the relative intensity of the three characteristic peaks highlighted in Figure 6a. The prominent peaks at ∼ 1.8 Å visible in Figure 6a stem from Mo–O distances, which can range between 1.7 and 2.5 Å.^81,82^ This distribution of distances is reflected in the shown reference PDFs. While the monomer only exhibits a peak at ∼ 1.8 Å, POMs of higher nuclearity also include Mo–O distances of up to 2.5 Å in length. This variation is typical for Mo-based POMs.^83^ Pairs of Mo atoms in our POM geometries take on distinct distances of ∼ 3.3 Å and ∼ 3.8 Å. The Mo–Mo reference lines in Figure 6a are matched with the experimentally observed peak positions of ∼ 3.3 Å and ∼ 3.8 Å (Figure 6b). When probing multiple cluster species and counterions simultaneously, the identification of more intricate PDF features of POMs can become challenging. For example, the coexistence of {Mo_36_} and β-{Mo_8_} may hinder reliable conclusions on the β-{Mo_8_} content in solution. Moreover, minor structural variations and thermal vibrations have to be considered as our PDF data probes a large ensemble. In summary, the following comparison between predicted and observed speciation will primarily hinge on the characteristic peaks highlighted in Figure 6a. For Mo–O peak intensities, the comparison also has to consider the intensity distribution up to 2.5 Å. Overall, different nuclearities should be clearly distinguishable, while the degree of protonation is not.

### Comparison of Experiment and Simulation

Now, X-ray total scattering results are directly compared to the speciation predictions from POMSimulator. Based on the predicted molecular ratios (Figure 4d–f), we constructed linear combinations of the simulated PDFs (Figure 6a). These PDFs are then scaled by refining an overall scale factor. The resulting R_w_ values, as well as the difference curves G­(r)_exp_-G­(r)_calc_, serve as indicators of how well the predicted PDF describes the experimental data. For pH = 1.6, individual simulated PDFs contributing to G­(r)_calc_ are also shown to emphasize the link between features in G­(r)_calc_ and the predicted speciation. Note that no structural parameters have been refined throughout the following analysis, which in turn prevents the determination of an error for the discussed peak positions.

For pH = 1.6, POMSimulator reliably predicts the POM speciation at ambient temperature, as evidenced by Figure 7a. All key features of the experimental PDF are accounted for by the calculated PDF. The peaks at ∼ 2.4 Å and ∼ 3.2 Å are caused by the metal–oxygen distances in Cl^–^ and Na^+^ hydration shells,^84–86^ as confirmed by the experimental reference PDF for NaCl_aq_ (2 M). The remaining mismatches below 7 Å can be attributed to our use of one molecular geometry per cluster type without additional structure refinement. Upon increasing the temperature to 75 °C, we expect a destabilization of {Mo_36_} in favor of {Mo_7_} and observe significant changes in the experimental PDF. Moreover, more pronounced differences between calculated and experimental PDF arise, as evidenced by Figure 7b and quantified by the R_w_-increase between Figure 7a and b. The experimental features between 5 and 10 Å are not fully described in Figure 7b. Instead, a close resemblance with the corresponding r-range of the G­(r)_exp_ in Figure 7a becomes apparent. This observation indicates that the actual molecular ratio of {Mo_36_} exceeds the predicted one. Nevertheless, the overall visual agreement between G­(r)_calc_ and G­(r)_exp_ is satisfying. Furthermore, the 75 °C prediction captures the relative intensities of the Mo–Mo peaks at ∼ 3.3 Å and ∼ 3.8 Å reasonably well, considering the presence of hydrated counterions.

**Figure 7:**
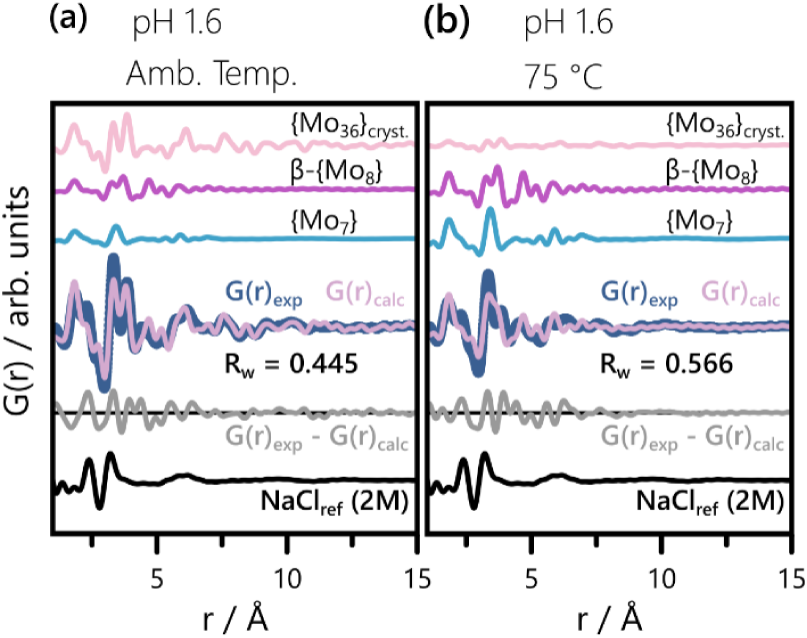
Comparison of the experimental and predicted PDFs at pH = 1.6 for (a) ambient temperature and (b) 75 °C. The contributing simulated PDFs, scaled according to the molecular ratios from Table S4, are depicted using the color code introduced in Figure 4. The experimental data, G­(r)_exp_, and predicted linear combination, G­(r)_calc_, give rise to the difference curves shown in light gray. The experimental PDF of an aqueous NaCl solution (2M) is added for reference.

Generally, Figure 7 underpins the reliability of POMSimulator between pH 1 and 2 regarding temperature-dependent trends but also highlights the intrinsic uncertainty of our approach regarding quantitative molecular ratios. Nonetheless, the hybrid experimental/computational approach shows to be capable of informing of this uncertainty. Contrasting experimental insight with predicted speciation can moreover deepen our understanding of gradual crystallization of h-MoO_3_ ^80^ observed for pH = 1.6 at 125 °C (Figure 5a and d). Although the predictive power of POMSimulator is limited to molecular species, it can help us deduce the underlying rational of this crystallization. For pH = 1.6, POMSimulator predicts a decrease from 56% {Mo_36_} at 25 °C to a negligible share of 2% {Mo_36_} remaining at 125 °C. Instead of {Mo_36_}, β­{Mo_8_} is predicted as most abundant for pH = 1.6 and 125 °C. The experimental data in Figure 5a points toward a low abundance of {Mo_36_} in “First” frame of the pH = 1.6 at 125 °C data set, indicated by the low intensity at ∼ 3.8 Å. Instead of the characteristic second Mo–Mo peak of {Mo_36_}, only a shoulder is visible in this “First” frame, which experimentally confirms the predicted destabilization of {Mo_36_} at 125 °C. However, instead of an equilibrium dominated by β-{Mo_8_}, {Mo_36_} destabilization results in h-MoO_3_ crystallization. This observation only occurred at pH = 1.6 and 125 °C, although large POMs get destabilized upon heating throughout the probed pH range. Accordingly, pH seems to play a crucial role in h-MoO_3_ formation. The impact of pH on cluster nuclearity is linked to condensation reactions.^15^ A decrease in pH shifts the corresponding equilibria toward a higher degree of condensation, reflected in an increased Mo/O ratio. Using this measure to compare {Mo_36_} (Mo_36_O_112_(H_2_O)_16_^8–^; Mo/O = 0.321) and β-{Mo_8_} (Mo_8_O_26_^4–^; Mo/O = 0.307) with h-MoO_3_ (Mo/O = 0.333) reveals that the predicted decomposition of {Mo_36_} into β-{Mo_8_} would entail a decrease in condensation degree, which is impeded by a low pH. Thus, {Mo_36_} destabilization combined with a high degree of condensation induced by the low pH are likely key driving forces behind the observed h-MoO_3_ crystallization. While the observed oxide formation reveals a decreased molybdate solubility at pH = 1.6 and 125 °C, more general insights into the complex link between POM fragmentation and solubility are beyond our framework and scope.

For pH = 3.4, we observe an overall good agreement between experimental and simulated data, as evidenced by Figure 8a. Moreover, R_w_ decreases with heating, which suggests increasing agreement between prediction and experiment. Visual inspection of Figure 8a reveals that this trend primarily reflects how well the peak at ∼ 3.3 Å is accounted for. At 125 °C, the intensity ratio of the experimental Mo–O (1.8 to 2.5) Å and Mo–Mo (∼ 3.3 Å) peaks is best described by the predicted speciation. Considering the presence of hydrated Cl^–^ in solution, Cl–O pairs (∼ 3.2 Å) also contribute to this ratio, although they are not considered in G­(r)_calc_. Decoupling the Cl–O contribution from the Mo–Mo peak at ∼ 3.3 Å is not possible, due to a lack of insight into the effect of hydrothermal conditions on the PDF of hydrated Cl^–^ and the close proximity of the two peak positions. To gain reliable insight, it is therefore important to complement the comparison of experimental and simulated PDFs (Figure 8a) with a direct assessment of the experimentally observed trend (Figure 8b).

**Figure 8:**
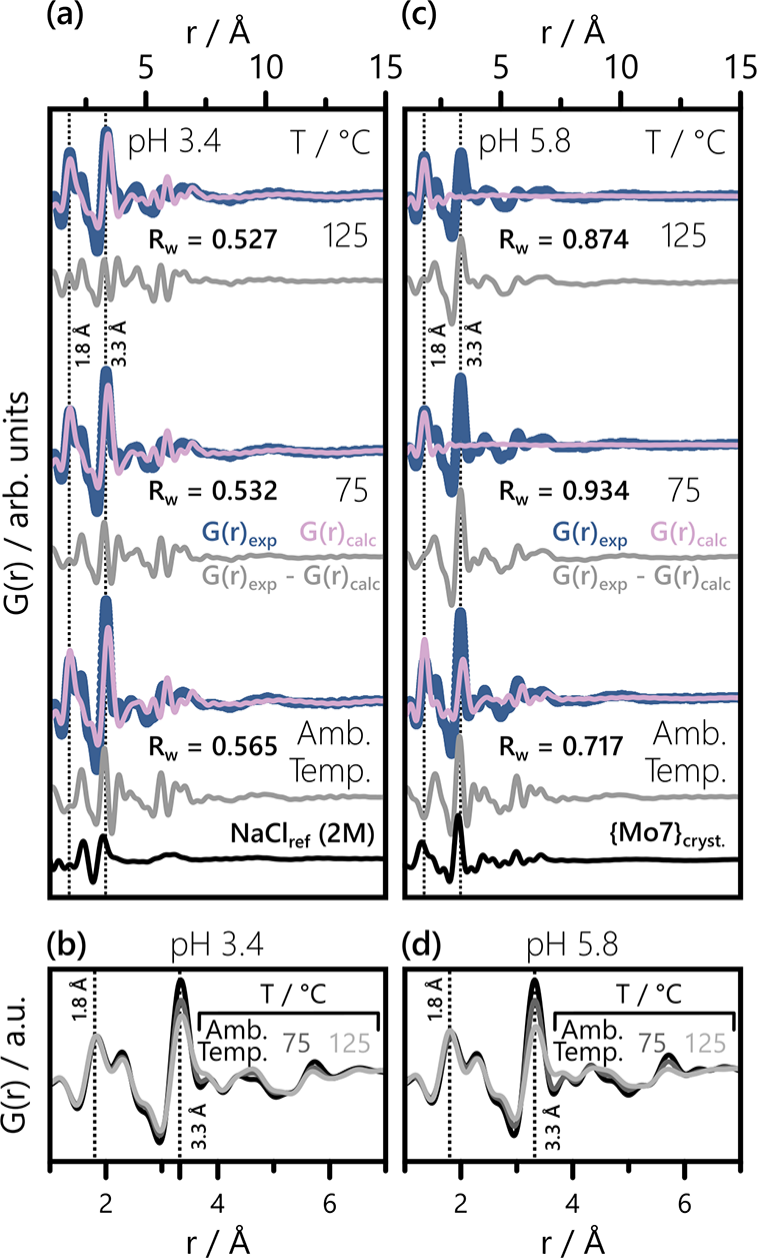
Comparison of the experimental and predicted PDFs at (a) pH = 3.4 and (c) pH = 5.8 across all three temperatures. The experimental data, G­(r)_exp_, and predicted linear combination, G­(r)_calc_, give rise to the difference curves shown in light gray. The experimental PDF of an aqueous NaCl solution (2M) and the calculated PDF of the solid-state {Mo_7_} structure are added for reference in (a) and (c), respectively. Direct comparison between the experimental PDFs at (b) pH = 3.4 and (d) pH = 5.8 is facilitated by overlaying the experimental data of all three temperatures directly on top of each other. Ambient temperature is shown in black, 75 °C in dark gray and 125 °C in light gray.

Comparing the experimental PDFs across temperatures reveals a decrease in intensity at ∼ 3.3 Å, as evidenced by in Figure 8b. Specifically, the intensity ratio between the peak intensities within 1.8 to 2.5 Å (Mo–O) and the ∼ 3.3 Å (Mo–Mo) peak drops. This indicates a decrease in average POM nuclearity, if temperature-induced changes in the Cl^–^ hydration shells are minor. Our experimental observations therefore point toward a gradual loss of {Mo_7_} in favor of smaller species, such as the monomer [MoO_4_]^2–^, when heating the pH = 3.4 sample.

However, this trend is not captured by our computational predictions, as evidenced by Figure 8a. In fact, the predicted average POM nuclearity changes little across the probed temperatures, as demonstrated by the side-by-side comparison of the simulated PDFs from Figure 8a in Figure S17. In contrast to our experimental data, the Mo–Mo/Mo–O ratio remains close to unchanged between the three predicated PDFs. Nonetheless, the general trend of our experimental results at pH = 3.4 agrees well with our qualitative prediction visualized in Figure 3: POMs of higher nuclearity get destabilized in favor of smaller species when heating is applied. The data in Figure 8b does not clearly indicate which smaller species are present upon heating. While the heat-induced decrease in intensity at ∼ 3.3 Å points toward a decreasing nuclearity, the increasing intensity ∼ 4.5 Å points toward multinuclear clusters instead of a coexistence of monomer and {Mo_7_} alone. This observation agrees with Raman results by Noack et al.,^87^ indicating the presence of bi- and trimolybdate species at 190 °C and [Mo^VI^] = 0.2 M. Nevertheless, the overall low intensity at ∼ 4.5 Å indicates a comparably low concentration of such smaller POMs. In general, we find that speciation prediction by POMSimulator is in itself consistent, but that the incorporation of PDF analysis into the study enables the identification of other minor species, enriching the description of the system.

Quantitative agreement between prediction and experiment is likely hindered by both computational and experimental uncertainties. This includes that we assumed a constant pH for our comparison, although temperature-induced p*K*_w_ changes (Figure 1d) influence the pH of our studied solutions. For example, Noack et al. observed pH changes from 5.4 to 5.8 when heating ammonium heptamolybdate solutions with [Mo^VI^] = 0.2 M from 20 to 190 °C.^87^ Similarly, uncertainties in the experimental temperature calibration could affect the comparison. Overall, the combined assessment of the pH = 3.4 experiment and prediction highlights that overarching trends in our predictions are reliable, which allows to derive valuable insight, even if quantitative mismatches appear.

At pH = 5.8, the prediction of POM speciation showed to be more challenging than at the lower two pH values. None of the three experimental PDFs is reasonably described by the calculated PDFs, as evidenced by Figure 8c. The difference curves in Figure 8c clearly exhibit PDF features matching the fingerprint of {Mo_7_} clusters, which sets them apart from the undescribed features in Figures 7 and 8a. Comparison with the simulated PDF of solid-state {Mo_7_} underpins the impression that POMSimulator underestimates the percentage of {Mo_7_} present under the probed conditions. While 57% of the speciation predicted for ambient temperature is comprised of {Mo_7_}, only 2% {Mo_7_} are predicted at 75 °C. According to POMSimulator, only monomer species should be present at 125 °C. Although these predictions significantly deviate from our experimental observations, a gradually decreasing share of {Mo_7_} is found in the experimental data as well, as the intensity at ∼ 3.3 Å decrease with increasing temperature in Figure 8d. As for the pH = 3.4 samples, this loss in Mo–Mo peak intensity points toward a decreasing average POM nuclearity, meaning a lower share of {Mo_7_} at higher temperatures. The predicted trend, therefore, primarily deviates from our experimental results in its magnitude.

The most likely cause for limited predictability at pH = 5.8 is the lack of direct experimental insight into the molecular geometries of species involved in {Mo_7_} formation. Accordingly, the initial geometries for POMs comprised of two to six metal atoms used in the POMSimulator workflow have to be guessed. While these building blocks provide a reasonable overall picture of the speciation, as discussed previously, their postulation may still affect the prediction of how [H_*x*_MoO_4_]^(2–*x*)–^ species transition into H_*x*_{Mo_7_} clusters. Thus, it seems reasonable that this kind of “frontier” region toward a neutral pH, where larger Mo clusters are not dominant, is less accurately described by the theoretical predictions. A deeper dive into the nature of these building blocks would likely require the inclusion of multiple isomers for each {Mo_*n*_} (*n* = 2 – 6) cluster. This approach would have to consider both transient species involved in the formation of larger anions and more stable isomers of these small building blocks, which more closely resemble larger experimentally identified clusters. Accounting for all potentially involved isomers would vastly increase the complexity of the reaction network, yielding simulations much more costly or even unfeasible. Yet again, it is the synergy between experiments and theory which gives a more complete depiction of the speciation.

All in all, the adapted POMSimulator workflow successfully captures qualitative trends of temperature-dependent cluster destabilization. Good agreement is reached for pH = 1.6, whereas the experimentally observed speciation at pH = 5.8 encounters issues regarding the prediction of smaller Mo clusters. However, combined with expert knowledge, the predicted speciation significantly accelerates experimental data analysis, as it provides a chemically informed reference point.

## Conclusions

The deep structural richness associated with pH-dependent polyoxometalate self-assembly and speciation prompts for the development and utilization of novel strategies to gain a better understanding of the underlying processes. In this work, we report, for the first time, the combination of a computational tool to predict the speciation of POMs with X-ray total scattering characterization techniques. This procedure has been applied to the speciation of molybdates under hydrothermal conditions, determining the outcome of self-assembly processes taking place at large pressures (100 bar) and at a wide range of temperatures (25 – 125 °C).

From the theoretical point of view, we highlight the fundamental importance of accounting for the effects of temperature and pressure in the parametrization of implicit solvation models, which strongly affect the predicted energies and, consequently, the speciation and speciation phase diagrams produced by POMSimulator. Moreover, the possibility of predicting speciation diagrams simplifies the nontrivial assignment of X-ray total scattering results, by providing a solid initial guess across the whole pH range. On the other hand, experimental insight into structural arrangements clarifies phenomena that cannot be properly modeled computationally, such as the crystallization of molybdenum oxide happening at pH = 1.6 and 125 °C. All in all, this work provides a robust foundation for further studies on polyoxometalate speciation beyond ambient conditions, confirming the synergy between experimental and theoretical methods to shed light into intricate chemical problems.

## Supplementary Material



## Notes

Solid-state POM structures and their atomic xyz coordinates, validation of the free energy calculation protocol, analysis of solvation parameter effects on electronic energies, validation of speciation model selection and averaging, extraction of molecular ratios from POMSimulator results, further details on h-MoO_3_ crystallization (PDF)

## #

Instituto de Micro y Nanotecnología (IMN CNM, CSIC), C/Isaac Newton 8, Tres Cantos, Spain
