# Supplementary material for: Uncovering Polyoxometalate Speciation in Hydrothermal Systems by Combining Computational Simulation with X‑ray Total Scattering
Source: J Am Chem Soc. 2025 Jun 17;147(26):22747–58. doi: 10.1021/jacs.5c04696 (PMC12232304; doi:10.1021/jacs.5c04696)
Supplement: Supplementary file 1 [file ja5c04696_si_001.pdf]

Supporting Information

Uncovering polyoxometalate speciation in hydrothermal systems by combining computational simulation with X-ray total scattering

Laura S. Junkers[1†], Diego Garay-Ruiz[2,6†], Jordi Buils[2,3], Rebecca S. Silberg[1], Guilherme B. Strapasson[1,4,5], Kirsten M. Ø. Jensen[1*], and Carles Bo[2,3*]

[1] Department of Chemistry, University of Copenhagen, Universitetsparken 5, 2100 Copenhagen, Denmark

[2] Institute of Chemical Research of Catalonia (ICIQ), Avinguda Països Catalans 16, 43007 Tarragona, Spain

[3] Departament de Química Física i Inorgànica, Universitat Rovira I Virgili (URV), Marcel·lí Domingo 1, Tarragona 43007, Spain

[4] Institute of Chemistry, University of Campinas, Rua Monteiro Lobato 270, Campinas SP 13083-862, Brazil

[5] Brazilian Synchrotron Light Laboratory, CNPEM, R. Giuseppe Máximo Scolfaro, 10000, Campinas SP 13083-100, Brazil

[6] Current address:
Instituto de Micro y Nanotecnología (IMN CNM, CSIC), C/Isaac Newton 8, Tres Cantos, Spain

[†]equal contributions

*corresponding authors

Contents

Additional experimental details ... 2

POM structures cut out from solid state ... 2

Further discussion ... 2

Section 1 - Validating the energy calculation strategy ... 2

Section 2 – Analyzing of ε-induced changes in electronic energy ... 2

Section 3 – Validation of speciation model choice and averaging ... 3

Section 4 – Extracting molecular ratios from speciation diagrams ... 3

Section 5 – Details on the formation of h-MoO_3 ... 3

Figures and Tables ... 4

General context ... 4

Experimental ... 14

References ... 20

Additional experimental details

POM structures cut out from solid state

To simulate PDFs of POM structures determined from single crystal crystallography, clusters were cut out from crystal structures of their salts: {Mo_7} was obtained from $Na_7(Mo_7O_{24})(OH)(H_2O)_{21}$,[1] β-{$Mo_8$} from $((CH_3)_3S)_4(Mo_8O_{26})$,[2] and {$Mo_{36}$} from $Na_8[Mo_{36}O_{112}(H_2O)_{16}] \cdot 58\ H_2O$.[3] The respective atomic coordinates are provided as supplementary xyz files. Figure S5 visualizes the three structures, compared to their DFT-derived counterparts.

Further discussion

Section 1 - Validating the energy calculation strategy

We calculated Gibbs free energies under consideration of temperature-dependent solvent properties following the approach schematically represented in Figure 2. This strategy roots in the assumption that the electronic energies E_{el} are affected by changes in ε and R_{solv} while the G_{corr} terms can be accurately derived from frequencies calculated under standard conditions.

We validated this cost-efficient approach with test calculations on a subset of five unprotonated POMs, {Mo_x} with x = [1, 2, 4, 6, 7], spanning a temperature range from 25 °C to 250 °C. For these molecules, we calculated the Gibbs free energies using the composite method detailed in Figure 2 and compared those results of full geometry re-optimizations and frequency re-calculations based on correct COSMO parameters. Figure S6 facilitates a direct comparison between the G_{corr} terms obtained through the composite-method and the full re-calculation with correct ε and R_{solv} values. Overall, the two sets of G_{corr} terms differ negligibly. G_{corr} shows to be independent from the solvent parameters throughout all test molecules except {Mo_7}, whose G_{corr} values are plotted in Figure S6(e). These deviations occur, since the low-lying vibrational modes of {Mo_7} fall right at the 20 cm^{-1} cut-off employed in ADF. Consequently, our text calculations overall show that neither the geometry nor the frequencies are significantly impacted by the temperature-induced solvent changes. Accordingly, geometries and vibrational normal modes determined under standard conditions can be used throughout our temperature range of interest (from 25 °C to 250 °C). Only the electronic energies E_{el} have to be recalculated to account for changed solvent parameters, which substantially limits the computational cost.

Section 2 – Analyzing of ε-induced changes in electronic energy

To determine the impact of the hybrid-approach proposed in Figure 2, we assessed the effect of re-calculating the electronic energies, E_{el}, with correct COSMO parameters. In contrast to Section 1, the following comparison takes place between values obtained through the proposed hybrid-approach (Figure 2) and values completely neglecting the temperature-dependent nature of ε and R_{solv}.

We chose the free energies of formation as measure of comparison across the probed temperatures. In line with both the formation constants reported experimentally and predicted by POMSimulator, we assumed formation reactions starting from $[MoO_4]^{2-}$ as a reference. We limited the assessment to the eight molecules for which Cruywagen[4] reported experimental formation constants (listed in Table S1).

Figure S7 gives an overview of the relative error between formation energies derived with and without recalculating E_{el} with correct solvent parameters. In other words, it compares the outcome of the approach detailed in Figure 2, ΔG*, with simply applying standard statistical thermodynamics to previously reported calculations based on a Large frozen core and standard gas-phase conditions (298.15 K, 1 atm), ΔG. The relative error was calculated as $100 \cdot (\Delta G - \Delta G^*) / \Delta G^*$.

Overall, the relative errors caused by neglecting temperature-dependent solvent properties increase significantly with temperature, as evidenced by Figure S7. Already at 75 °C, most formation energies exhibit relative errors of around 2 % higher than obtained for 25 °C. This effect increases towards 175 °C, where all of the formation energies have relative errors above 4 %, going up to around 8 % for $H_{32}Mo_{36}O_{128}^{8-}$. While the error patterns at each temperature in Figure S7 visually resemble one another, the relative error of a given

molecule does not change linearly with temperature. No simple correlation between the charge or size of a given molecule and the respective errors is observed.

Overall, these results demonstrate that neglecting temperature-induced changes in ε and R_{solv} results in significant energy errors, which cannot be reasonably approximated by simple extrapolation approaches.

Section 3 – Validation of speciation model choice and averaging

In choosing a speciation model for our POMSimulator predictions beyond standard conditions, we aimed for striking a balance between comparability with previously reported results under standard conditions and robustness by accounting for the variability between models. Since our study involves the same system over several temperatures, similar, yet distinct sets of formation constants need to be compared. We can therefore either choose the same model throughout all four data sets or individually determine the most suitable models at each temperature.

A common reference model facilitates comparison between the reaction networks at different temperatures as we would work with a common set of reactions. However, the agreement of calculated formation constants of a given model with experimental references significantly varies between the four temperature sets. This is evidenced by Figure S8, which shows that the six models with the lowest RMSE in the 25 °C set have drastically different RMSE values for the other temperatures. Across the bench, the best models at 25 °C exhibit high RMSE values at elevated temperatures. This prevents the use of a common speciation model.

Extracting meaningful insight from the comparison of predictions across temperatures requires a certain robustness of the chosen speciation model. Such robustness is not possible when strictly following the RMSE-based selection approach, since even the two best models can already differ vastly. This is the case for the 75 °C set, where the phase diagrams of the best and second-best model do not resemble each other, as evidenced by Figure S9(a) and (b). Nonetheless, both models have similar RMSE values, as demonstrated by Table S3. To account for this variability among speciation models, we worked with average speciation of the **n** best models and tested **n** = 10, 25, or 50. The phase diagrams obtained for the 75 °C set based on these approaches to speciation model choice are compared in Figure S9. In contrast to the phase diagram of the best and second-best model (Figure S9(a) and (b)) the phase diagrams obtained from averaged models, labelled as avg10, avg25 and avg50 in Figure S9(c)-(e), resemble each other. Particularly the avg25 and avg50 diagrams look alike. This indicates that averaging over 25 models yields increased robustness compared to 10 models. On the other hand, using 50 models does not significantly improve the result. Aiming to average as few models as possible to retain a high agreement with experimental formation constants and limit computational costs, we use the avg25 predictions for our experimental data analysis.

Section 4 – Extracting molecular ratios from speciation diagrams

We extracted molecular ratios from the predicted speciation diagrams by dividing the predicted Mo% values by the number of Mo atoms in the respective POM structure, *e.g.*, 7 for $\{Mo_7\}$. In this step, all species making up less than 1 % of the total Mo^{VI} amount (Mo% < 1) were excluded, reflecting sensitivity limits of our experimental data. Subsequent normalization by the sum of all contributions from the remaining species yielded the molecular ratios listed in Table S4 and visualized in Figure 4(d)-(f). These ratios served as scale factors for our PDF data analysis.

Section 5 – Details on the formation of h-MoO_3

At 125 °C, we observe the formation of a crystalline phase from the sample solution with pH = 1.6, as evidenced by the long correlation length of the PDF obtained after 3 min of heating to 125 °C ("Last"), shown in Figure 5(a). Here, we dive further into the analysis of this crystalline phase and its formation. The formed structure was identified as hexagonal MoO_3 (h-MoO_3,[5] visualized in Figure S10(a)), as evidenced by the phase matching in reciprocal space shown in Figure S10(b). PDF refinements underpin this observation,

as demonstrated in Figure 5(d) and Figure S10(c). However, below 6 Å significant deviations from the h-MoO_3 model are visible in Figure S10(c), as shown in more detail in Figure S10(d), which depicts the same refinement. These mismatches indicate the presence of remaining POM species, as evidenced by the resemblance of the difference curve in Figure S10(d) and the "First" frame of the pH = 1.6, T = 125 °C data set shown in Figure 5(a). In summary, these results show that h-MoO_3 crystallizes from the highly acidic Na_2MoO_4 solution (pH = 1.6) at 125 °C and POM species with a correlation length of 6 Å remain present up until 3 min into the crystallization process. Accordingly, changing the fit range of the refinement in Figure 10(c) and (d) to include 6-100 Å, as done in Figure 5(d), results in a significantly improved R_w value.

Moreover, the Pearson Correlation matrix of the data set exhibits a significant color gradient, as is directly compared to the correlation matrices of the other data sets in Figure S11 and most visible in Figure S14. This observation confirms that the probed crystallization process is gradual and not completed by the time our measurement ended.

Figures and Tables

General context

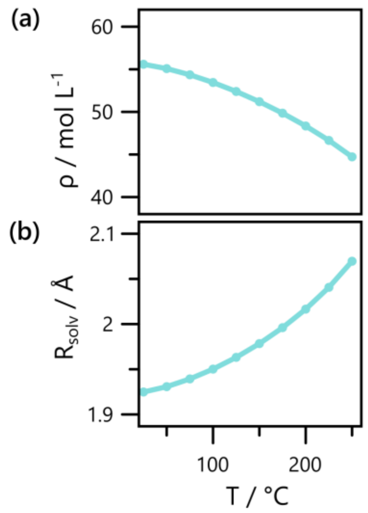

Figure S1. (a) Density of water, ρ, plotted against temperature up until 250 °C, assuming a pressure of 100 bar.[6] (b) Solvent radius calculated based on the density using Equation S1.

Equation S1. Relation between density of water and the solvent radius R_{solv} used in COSMO (compare Figure S2(a)), based on the molecular weight M_w and Avogadro's constant.

$$\rho = \frac{m}{V} = \frac{M_w}{N_A}\left(\frac{4}{3}\pi(R_{solv})^3\right)^{-1} \rightarrow R_{solv} = \left(\frac{3}{4\pi}\frac{M_w}{\rho N_A}\right)^{1/3}$$

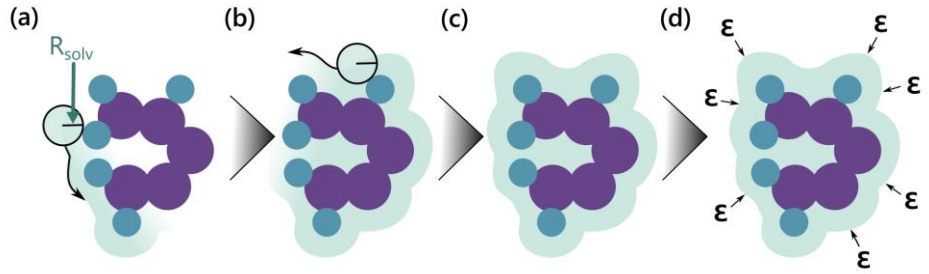

Figure S2. Schematic depiction of the continuous solvent model, COSMO,[7] applied in this study. (a) A spherical probe defined by its radius, R_{solv}, derived from density of water by Equation S1, is used to (b)-(c) determine the surface of the molecule accessible by the solvent. (d) Using this surface and the dielectric constant of the solvent, the influence of the solvent on the molecule is then modelled.

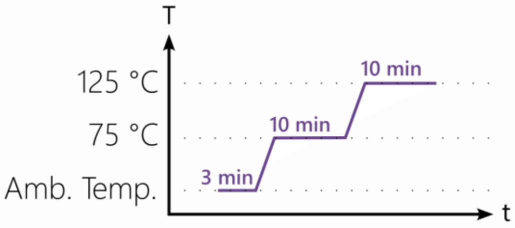

Figure S3. Schematic representation of the temperature profiles used for the X-ray total scattering experiments.

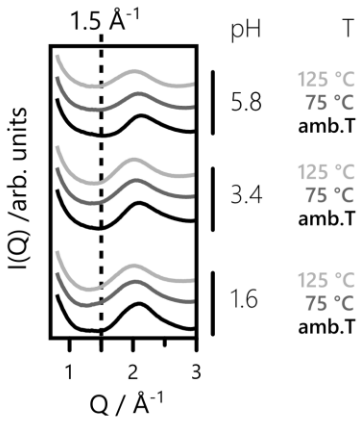

Figure S4. Overview over the first I(Q) patterns of the nine total scattering data sets. A vertical line at 1.5 Å^{-1} indicates the common lower limit (Q_{min}) chosen for the Fourier transforms to minimize the impact of SAXS contributions on the PDFs.

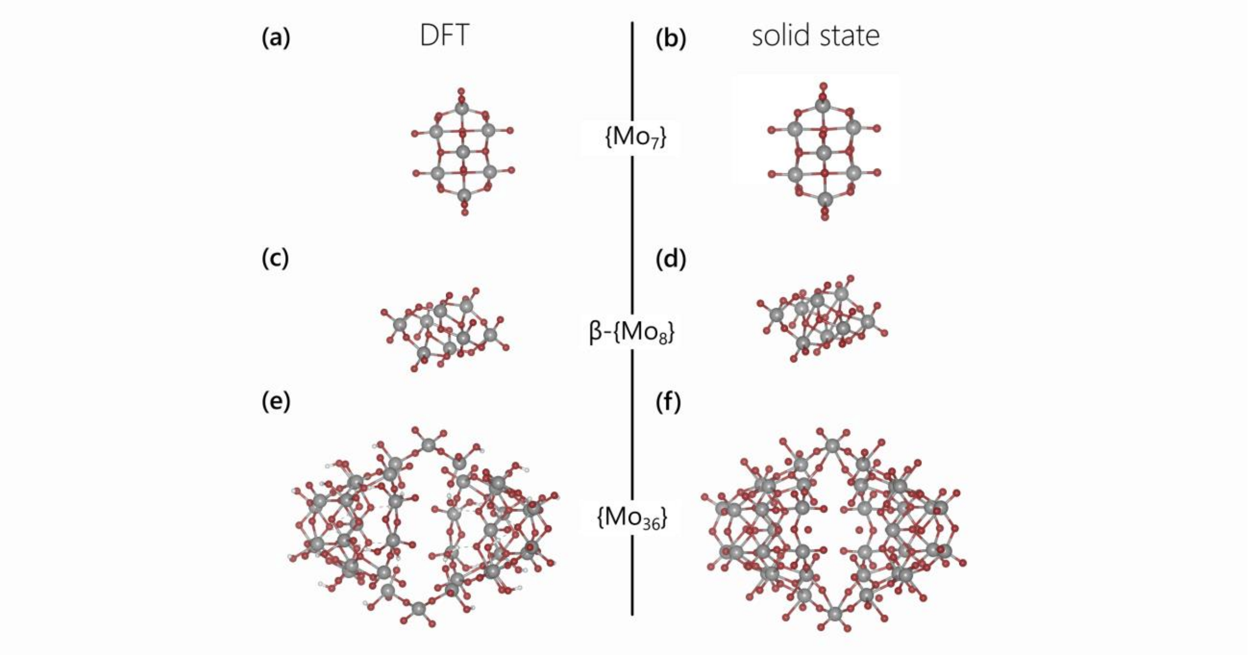

Figure S5. Side-by-side comparison of the DFT-derived molecular geometries of Mo7O24_0H, Mo8O26_0H and Mo36O128_32H with corresponding solid-state cut-outs of $\{Mo_7\}$, $\{Mo_8\}$ and $\{Mo_{36}\}$ from experimentally determine crystal structures (as detailed above). (a) DFT-derived Mo07O24_0H cluster structure. (b) Solid-state cut-out of $\{Mo_7\}$.[1] (c) DFT-derived Mo08O26_0H cluster structure. (d) Solid-state cut-out of β-$\{Mo_8\}$.[2] (e) DFT-derived Mo36O128_32H cluster structure. (f) Solid-state cut-out of $\{Mo_{36}\}$.[3] The respective PDFs are compared in Figure 6(a) and Figure S16(a), where calculated patterns of the POMs structures determined *via* single crystal crystallography are depicted in black (labelled as "cryst.") and the PDFs of DFT-derived structures shown in various colors.

Computational

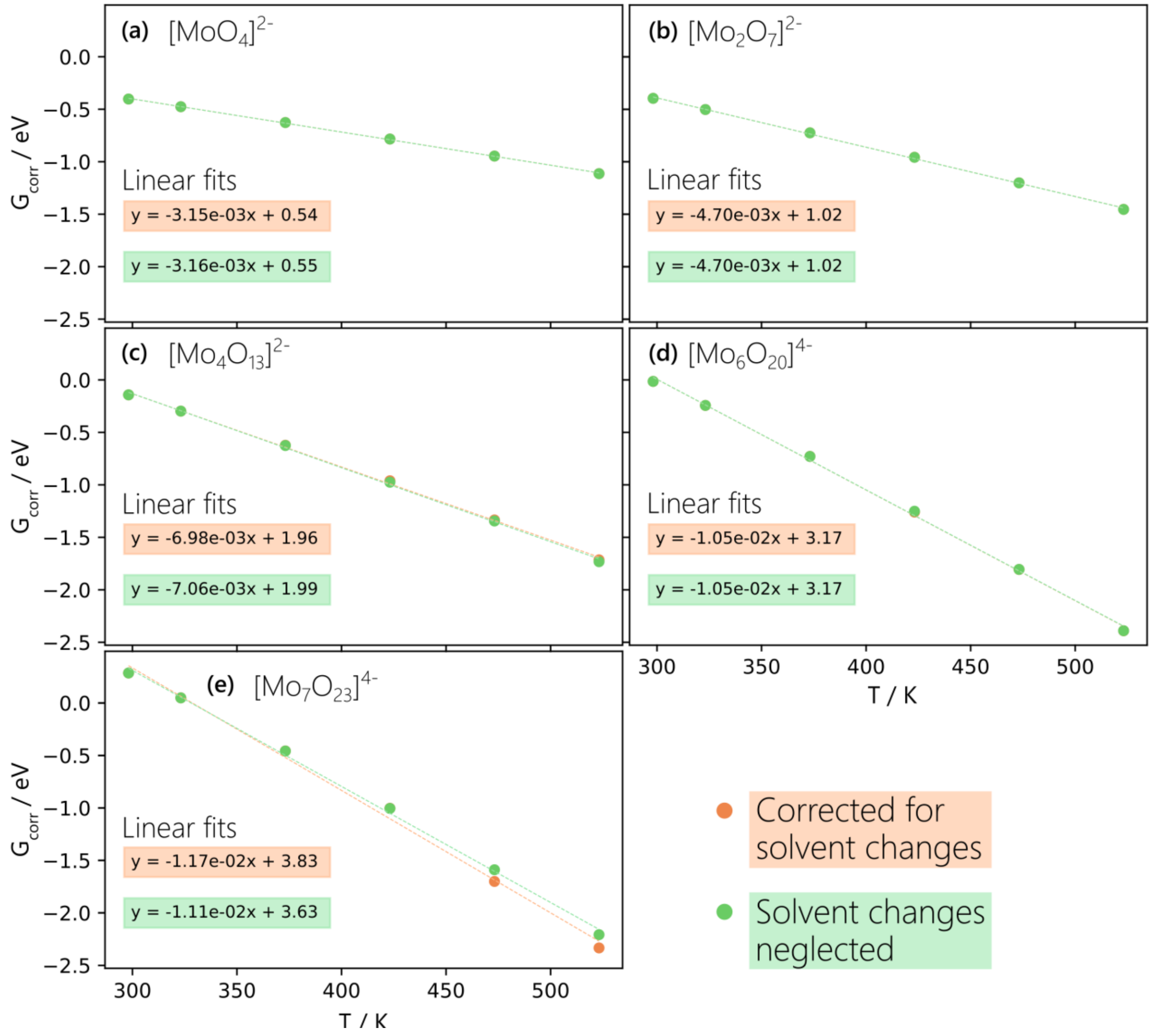

Figure S6. Comparison of the G_{corr} terms obtained through two calculation strategies between 298.15 K and 523.15 K (25 °C and 250 °C). The results of the composite method, schematically shown in Figure 2, are denoted as "solvent changes neglected" and depicted in green. The G_{corr} terms derived through full re-optimizations and frequency re-calculations with correct COSMO parameters are denoted as "corrected for solvent changes" and depicted in orange. The linear functions obtained from fitting both data sets are shown in green and orange boxes, respectively. The results for all five test molecules are shown: (a) $[MoO_4]^{2-}$, (b) $[Mo_2O_7]^{2-}$, (c) $[Mo_4O_{13}]^{2-}$, (d) $[Mo_6O_{20}]^{4-}$, (e) $[Mo_7O_{23}]^{4-}$.

Table S1. Overview over the nine experimental formation constants, K_f, by Cruywagen[4] used as input for POMSimulator, as well as their temperature-adjusted counterparts, derived based on the relation: $logK_f(T) = -RT\Delta G_r$. The temperature-adjusted constants are labelled as $K_f(T)$ with T equaling the temperature of the respective POMSimulator run. The experimental values were obtained under standard conditions (298.15 K).

Molecules	K_f(298.15 K) by Cruywagen[4]	K_f(348.15 K)	K_f(398.15 K)	K_f(448.15 K)
$HMoO_4^-$	3.55	3.04	2.66	2.36
H_2MoO_4	7.22	6.18	5.41	4.80
$Mo_7O_{24}^{6-}$	52.81	45.23	39.55	35.13
$HMo_7O_{24}^{5-}$	57.40	49.16	42.98	38.19
$H_2Mo_7O_{24}^{4-}$	60.97	52.21	45.65	40.56
$Mo_8O_{26}^{4-}$	71.19	60.97	53.31	47.36
$HMo_8O_{26}^{3-}$	73.07	62.58	54.72	48.61
$H_{32}Mo_{36}O_{128}^{8-}$	346.5	296.7	259.5	230.5

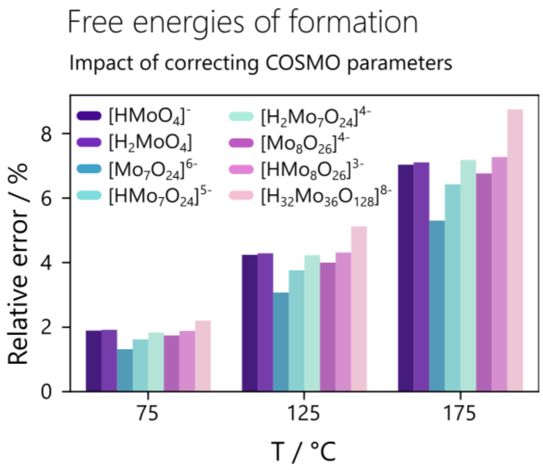

Figure S7. Overview over the relative errors of formation energies of various POM species caused by not accounting for the temperature-dependent water properties, derived as detailed in Section 2. To assess temperature effects, data for 75 °C, 125 °C, and 175 °C is shown.

Table S2. List of the Gibbs free energies, G, corrected for ε and R_{solv} changes based on the strategy visualized in Figure 2. The listed Gibbs free energies function as input for the discussed POMSimulator predictions. In addition to the 73 considered molecules, four variations of water were included in the POMSimulator input. Note that the 175 °C data set was only used for the energy comparison in Figure S7 and the model choice comparison in Figure S8.

	G (hartree)			
**Molecule\ T**	**25 °C**	**75 °C**	**125 °C**	**175 °C**
H2O	-0.52201642	-0.52460646	-0.52786986	-0.53090577
H3O	-0.45386698	-0.45624196	-0.45920802	-0.46177327
H4O2	-1.04203304	-1.04632097	-1.05178626	-1.0569195
H5O2	-0.99230218	-0.99587069	-1.00040992	-1.00454019
Mo01O04-0H	-1.76054026	-1.76430038	-1.76905671	-1.77307757
Mo01O04-1H	-1.75706263	-1.76204048	-1.76838293	-1.77424703
Mo01O04-2H	-1.72288831	-1.7283565	-1.73533832	-1.74191354
Mo01O06-6H	-2.74736926	-2.75463715	-2.76408235	-2.77310142
Mo01O06-7H	-2.69315357	-2.70024553	-2.70945713	-2.71814055
Mo01O06-8H	-2.61204351	-2.61870295	-2.62732694	-2.63513839
Mo02O07-0H	-2.99668186	-3.00328728	-3.01177327	-3.01949254
Mo02O07-1H	-2.96505739	-2.97252854	-2.98212339	-2.99112955
Mo02O07-2H	-2.92438642	-2.93218889	-2.94210964	-2.9515982
Mo02O10-6H	-4.4859264	-4.49443379	-4.50562406	-4.51613658
Mo02O10-7H	-4.46327552	-4.47238004	-4.48436451	-4.49590318
Mo02O10-8H	-4.42689473	-4.43662388	-4.44941129	-4.46177777
Mo03O10-0H	-4.20305542	-4.21198563	-4.22356242	-4.23431345
Mo03O10-1H	-4.16607369	-4.1758651	-4.18849811	-4.20042731
Mo03O10-2H	-4.12490599	-4.13508688	-4.14820954	-4.1606703
Mo03O14-6H	-6.2288461	-6.2368958	-6.24759677	-6.25686676
Mo03O14-7H	-6.25259568	-6.26256723	-6.27577452	-6.28800057
Mo03O14-8H	-6.22215099	-6.23342336	-6.24835004	-6.26258661
Mo04O13-0H	-5.42092468	-5.43130816	-5.44487573	-5.45767954
Mo04O13-1H	-5.37056294	-5.38150545	-5.39578376	-5.40944617
Mo04O13-2H	-5.31434473	-5.32562507	-5.34030706	-5.35438094
Mo04O18-6H	-7.98654741	-7.99476812	-8.00566238	-8.01393472
Mo04O18-7H	-7.93984918	-7.95141513	-7.96291515	-7.97408244
Mo04O18-8H	-7.99423868	-8.00541109	-8.02028336	-8.03375645
Mo05O16-0H	-6.5910516	-6.60264085	-6.61793198	-6.63252392
Mo05O16-1H	-6.56250139	-6.57497743	-6.59141981	-6.60731979
Mo05O16-2H	-6.47298727	-6.48589997	-6.50288563	-6.51932961
Mo05O17-0H	-7.16604085	-7.17649502	-7.19029542	-7.20255393
Mo05O17-1H	-7.15879908	-7.17058461	-7.18613806	-7.20065893
Mo05O17-2H	-7.12572334	-7.13820328	-7.15469951	-7.17050531
Mo05O22-6H	-9.59710394	-9.60409346	-9.60927411	-9.61214322
Mo05O22-7H	-9.65722731	-9.66660345	-9.67497209	-9.68197132

	G (hartree)			
**Molecule\ T**	**25 °C**	**75 °C**	**125 °C**	**175 °C**
Mo05O22-8H	-9.67857304	-9.69002260	-9.70120716	-9.71177762
Mo06O20-0H	-8.38313737	-8.39523888	-8.41130881	-8.42595830
Mo06O20-1H	-8.35474590	-8.36794857	-8.38541817	-8.40187621
Mo06O20-2H	-8.31655127	-8.33041025	-8.34878875	-8.36649902
Mo06O27-11H	-11.87696371	-11.88950593	-11.90159653	-11.91284880
Mo06O27-12H	-11.91359248	-11.92802053	-11.94263773	-11.95713271
Mo06O27-13H	-11.93170109	-11.94857404	-11.96624609	-11.98440958
Mo06O27-14H	-11.92784370	-11.94621774	-11.96585200	-11.98653576
Mo07O23-0H	-9.58812966	-9.60180288	-9.62002038	-9.63688300
Mo07O23-1H	-9.56062822	-9.57568647	-9.59572042	-9.61477396
Mo07O23-2H	-9.50387756	-9.51985694	-9.54109969	-9.56159738
Mo07O24-0H	-10.10645397	-10.11621208	-10.12935391	-10.14008866
Mo07O24-1H	-10.10112250	-10.11308667	-10.12917314	-10.14346729
Mo07O24-2H	-10.08180508	-10.09577711	-10.11449929	-10.13194060
Mo07O31-12H	-13.64376405	-13.65843317	-13.67245218	-13.68530501
Mo07O31-13H	-13.63554074	-13.65135065	-13.66716579	-13.68257009
Mo07O31-14H	-13.64040611	-13.65821935	-13.67669669	-13.69552113
Mo08O26-0H	-10.80275833	-10.81775046	-10.83784562	-10.85666797
Mo08O26-1H	-10.76693818	-10.78312686	-10.80479906	-10.82558654
Mo08O26-2H	-10.71702150	-10.73403163	-10.75679412	-10.77896204
Mo08O35-13H	-15.34270915	-15.35666432	-15.36958312	-15.38086108
Mo08O35-14H	-15.34784635	-15.36426513	-15.38036622	-15.39579806
Mo08O35-15H	-15.36753708	-15.38610429	-15.40516420	-15.42428576
Mo09O37-14H	-16.07396028	-16.09290018	-16.11862412	-16.14229472
Mo09O37-15H	-16.06867947	-16.08913506	-16.11693183	-16.14320032
Mo09O37-16H	-16.05837409	-16.08060207	-16.11071911	-16.13974788
Mo09O37-17H	-16.01601387	-16.03893319	-16.06995616	-16.10028294
Mo09O37-18H	-15.97256535	-15.99660607	-16.02910064	-16.06110112
Mo09O37-19H	-15.92192720	-15.94674204	-15.98025641	-16.01338195
Mo09O38-14H	-16.59317351	-16.60910275	-16.63093210	-16.64946736
Mo09O38-15H	-16.59051478	-16.60822221	-16.63237855	-16.65394549
Mo09O38-16H	-16.59313321	-16.61336835	-16.64088068	-16.66640739
Mo09O38-17H	-16.56077250	-16.58228231	-16.61148345	-16.63925305
Mo09O38-18H	-16.53908201	-16.56257554	-16.59436294	-16.62509557
Mo09O38-19H	-16.50469656	-16.52868282	-16.56112081	-16.59282125
Mo18O65-16H	-27.30322720	-27.33869933	-27.38707271	-27.43380261
Mo18O65-17H	-27.30533632	-27.34225937	-27.39248171	-27.44148869
Mo18O65-18H	-27.27548581	-27.31325213	-27.36467631	-27.41521834
Mo36O128-32H	-53.53908800	-53.59768049	-53.67856777	-53.75777445

Molecule\ T	G (hartree) 25 °C	75 °C	125 °C	175 °C
Mo36O128-33H	-53.48200125	-53.55057395	-53.64456834	-53.73712910
Mo36O128-34H	-53.49140566	-53.55601020	-53.64487327	-53.73269447

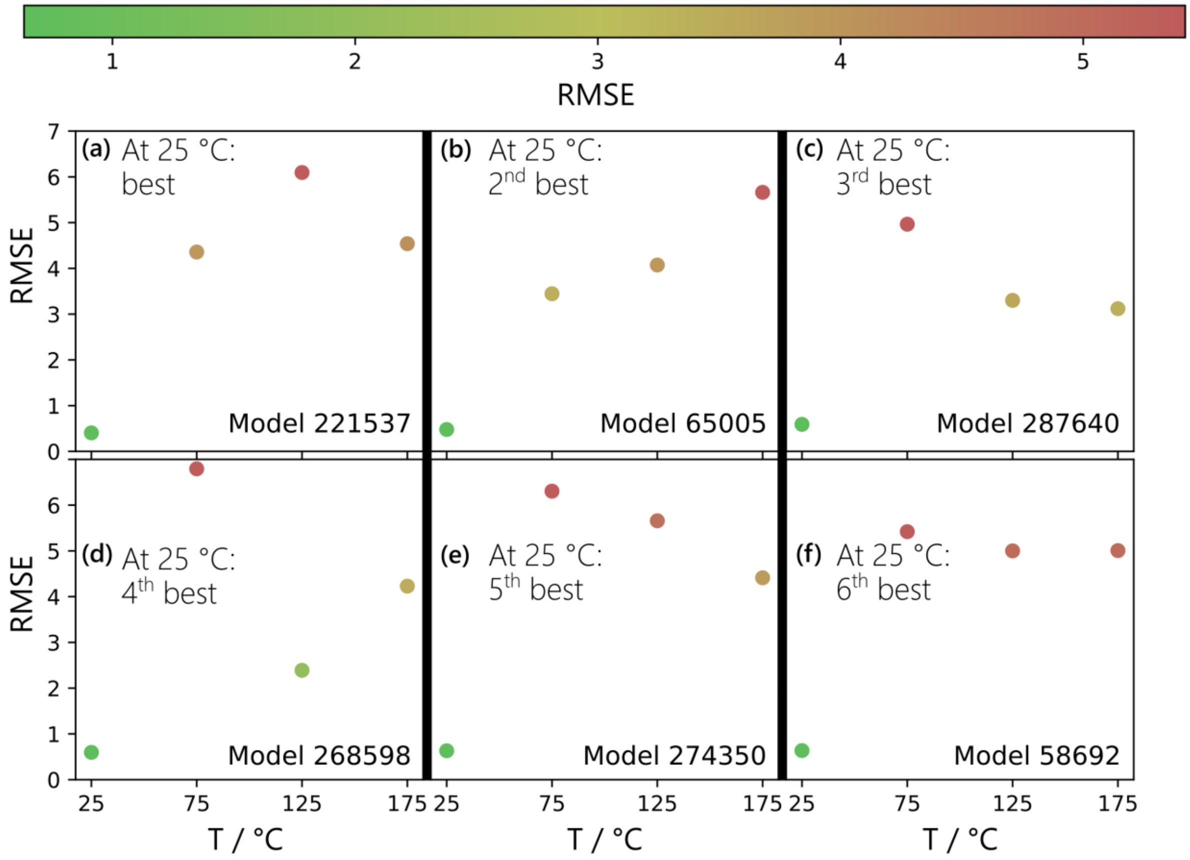

Figure S8. Assessment of how well the six best speciation models for the 25 °C set, (a)-(f), according to their RMSE, agree with experimental formation constants across the studied temperatures. To facilitate readability, RMSE is both color coded and plotted along the y-axis.

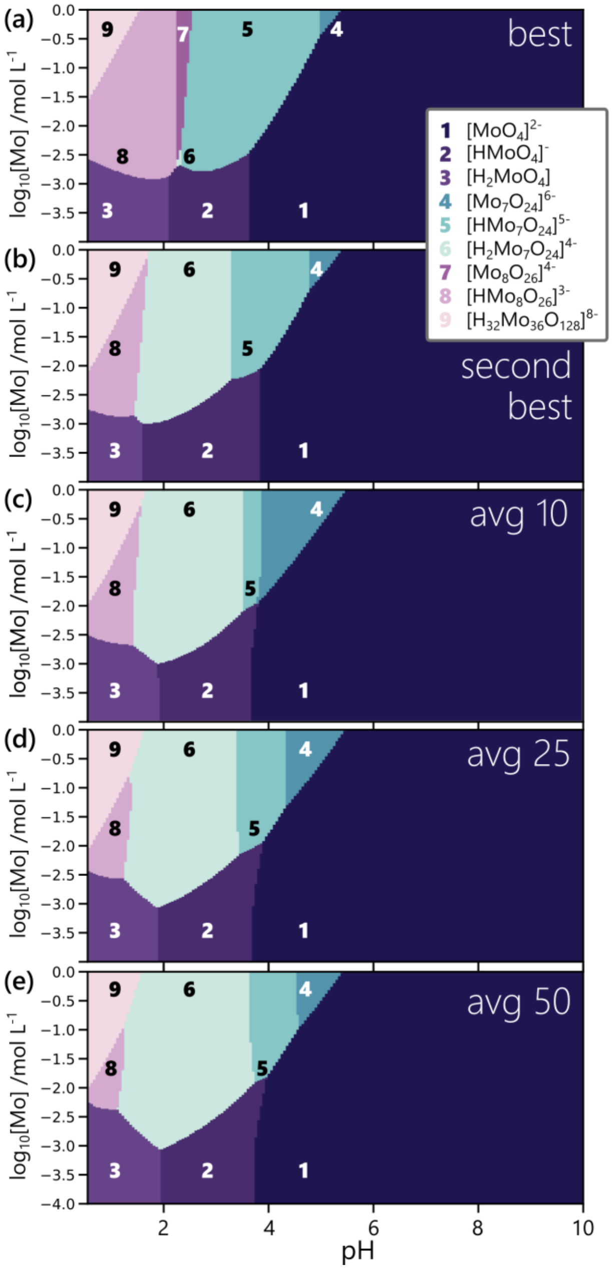

Figure S9. Comparison of phase diagrams of the 75 °C system based on five different approaches of speciation model choice, including (a) the best model regarding RSME, (b) the second-best model following this metric, (c) an average of the 10 best models, (d) an average of the 25 best models, and (e) an average of the 50 best models. Each speciation phase diagram represents a stacked representation of numerous speciation diagrams like the one depicted in Figure 1(b), differing in their Mo^{VI} concentration, [Mo]. This concentration is logarithmically plotted along the y-axis against the pH on the x-axis. The cluster species formed by the highest amount of Mo at each [Mo] and pH combination is encoded by color. Numbers were added to each phase diagram to facilitate the use of this color code.

Table S3. Comparison of the RMSE values obtained of the two best speciation models for each temperature. These RMSE values are obtained from linear regressions between predicted and experimental[4] formation constants.[8]

Temperature	RMSE of "best" model	RMSE of "second best" model
298.15 K	0.404299	0.478566
348.15 K	0.479004	0.492632
398.15 K	0.41195	0.436327
448.15 K	0.623773	0.625861

Table S4. Molecular ratios for all predicted species, derived from the predicted speciation as detailed in Section 4 and visualized in Figure 4(d)-(f).

Molecules	T = 298.15 K pH			348.15 K pH			398.15 K pH		
	1.6	3.4	5.8	1.6	3.4	5.8	1.6	3.4	5.8
MoO_4^{2-}	-	-	0.4257	-	-	0.9699	-	0.0675	1.0000
$HMoO_4^-$	-	-	-	-	-	0.0125	-	-	-
H_2MoO_4	-	-	-	-	-	-	-	-	-
$Mo_7O_{24}^{6-}$	-	0.0271	0.4142	-	0.1120	0.0176	-	0.1739	-
$HMo_7O_{24}^{5-}$	-	0.3228	0.1602	-	0.4367	-	0.1403	0.6640	-
$H_2Mo_7O_{24}^{4-}$	0.2099	0.5572	-	0.4928	0.4145	-	0.3310	0.9465	-
$Mo_8O_{26}^{4-}$	0.0650	0.0929	-	0.1493	0.0368	-	0.1855	-	-
$HMo_8O_{26}^{3-}$	0.1691	-	-	0.2529	-	-	0.3222	-	-
$H_{32}Mo_{36}O_{128}^{8-}$	0.5560	-	-	0.1051	-	-	0.0210	-	-

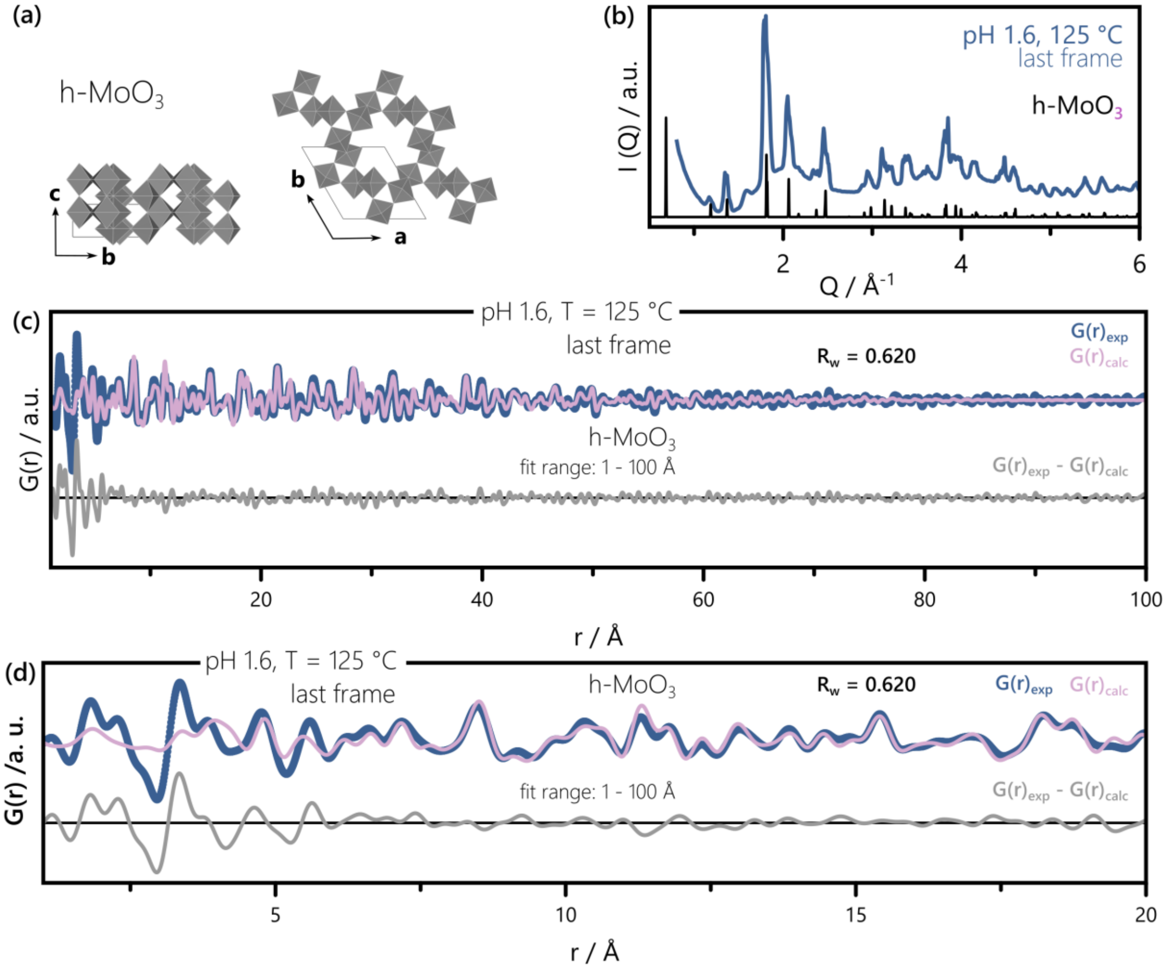

Figure S10. (a) Visualization of the h-MoO_3[5] crystal structure along two axes. MoO_x polyhedra are visualized in light gray. (b) Phase matching in I(Q) based on the last frame of the pH = 1.6, T = 125 °C data set, obtained after 3 min of heating to 125 °C. The experimental data is shown in blue and the h-MoO_3 reference pattern in black. (c)-(d) Structural refinement of h-MoO_3 to the last PDF of the pH = 1.6, T = 125 °C data set with a fit range from 1 to 100 Å. The two plots only differ in the depicted r-range. The corresponding fit parameter results are listed in Table S5.

Table S5. Refined parameters obtained by fitting h-MoO_3[5] to the last frame of the pH = 1.6, T = 125 °C data set, obtained after 3 min of heating to 125 °C. Two fit ranges were used, from 1 to 100 Å and from 6 to 100 Å.

Parameter	FigureS10(c) and (d)	Figure 5(d)
**Fit range / Å**	**1 – 100**	**6 – 100**
Scale factor	0.3604	0.3052
Particle size / Å	134	184
a, b	10.6647	10.6627
c	3.7192	3.7197
Common U_{iso} for Mo atoms	0.0098	0.0099
**R_w**	**0.620**	**0.414**

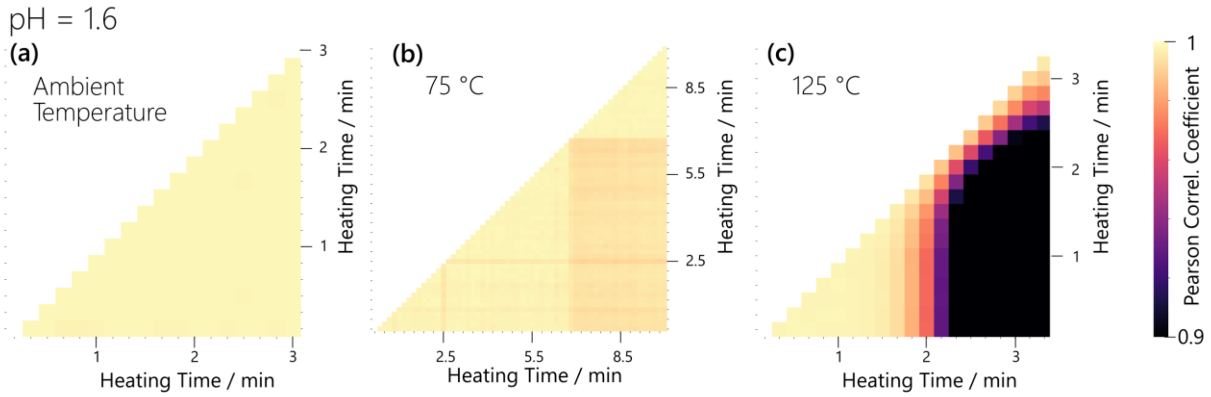

Figure S11. Pearson correlation matrices for the experimental data at pH = 1.6 at (a) ambient temperature, (b) 75 °C, (c) 125 °C, visualizing the similarity of all possible pairs of frames in the data set. Dark purple and yellow stand for low and high Pearson Correlation coefficients, respectively. Two identical PDFs yield a coefficient of 1 and complete lack of similarity yields a coefficient of 0. A common color code is used for comparability.

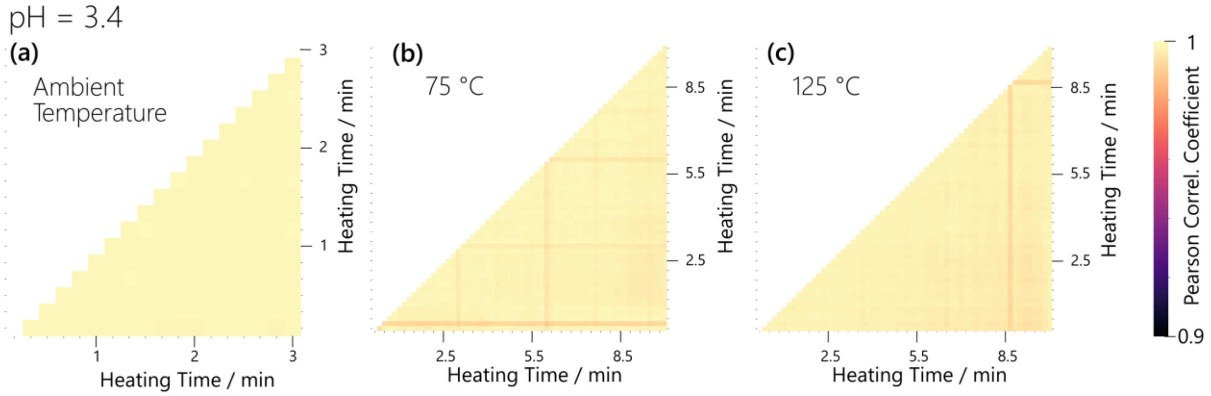

Figure S12. Pearson correlation matrices for the experimental data at pH = 3.4 at (a) ambient temperature, (b) 75 °C, (c) 125 °C, visualizing the similarity of all possible pairs of frames in the data set. Dark purple and yellow stand for low and high Pearson Correlation coefficients, respectively. Two identical PDFs yield a coefficient of 1 and complete lack of similarity yields a coefficient of 0. A common color code is used for comparability.

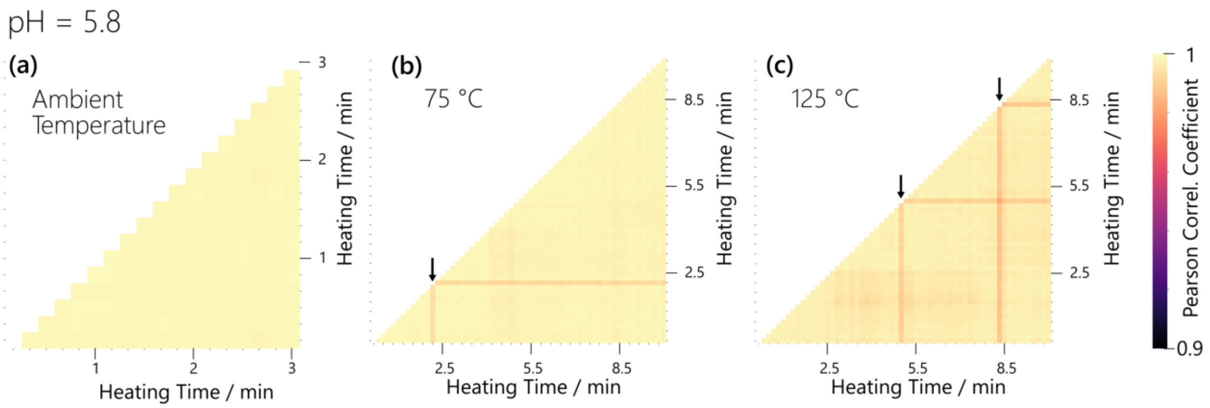

Figure S13. Pearson correlation matrices for the experimental data at pH = 5.8 at (a) ambient temperature, (b) 75 °C, (c) 125 °C, visualizing the similarity of all possible pairs of frames in the data set. Dark purple and yellow stand for low and high Pearson Correlation coefficients, respectively. Two identical PDFs yield a coefficient of 1 and complete lack of similarity yields a coefficient of 0. A common color code is used for comparability. Vertical arrows highlight examples of frames differing from their neighboring frames. Those frames are further assessed in Figure S15.

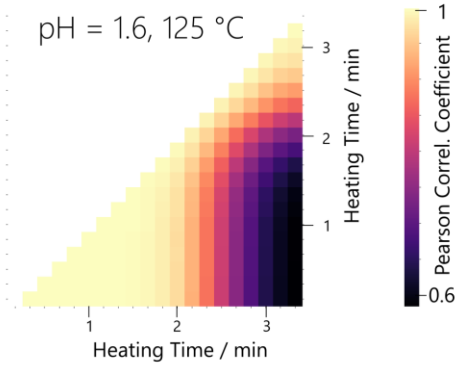

Figure S14. Pearson correlation matrices for the experimental data at pH = 1.6, T = 125 °C, based on an individual color code to further emphasize the gradual nature of the observed crystallization. Dark purple and yellow stand for low and high Pearson Correlation coefficients, respectively. Two identical PDFs yield a coefficient of 1 and complete lack of similarity yields a coefficient of 0.

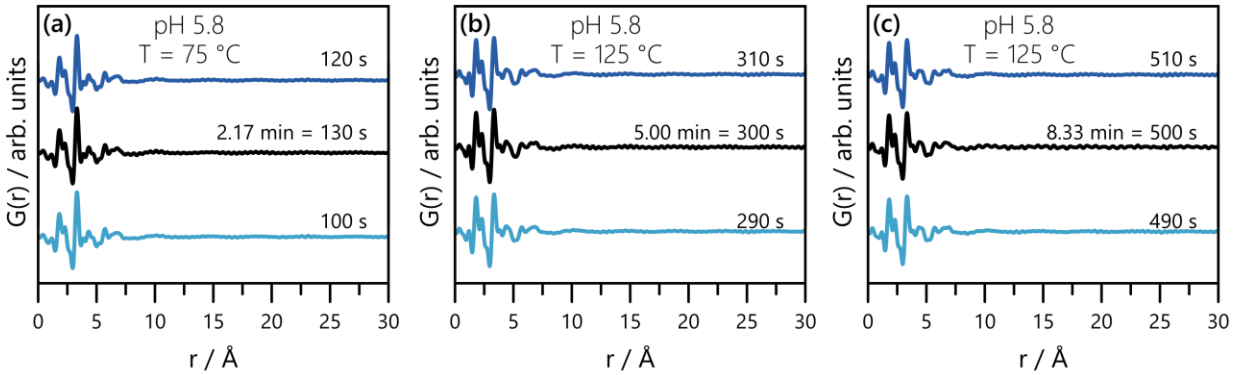

Figure S15. Comparison of the frames differing from their neighboring frames, highlighted by vertical arrows in Figure 5(e) and S13, corresponding to data collected at pH = 5.8 after (a) 2.17 min at 75 °C, (b) 5.00 min at 125 °C, and (c) 8.33 min at 125 °C. The "different" frame is shown in black with the two neighboring frames shown in blue above and below.

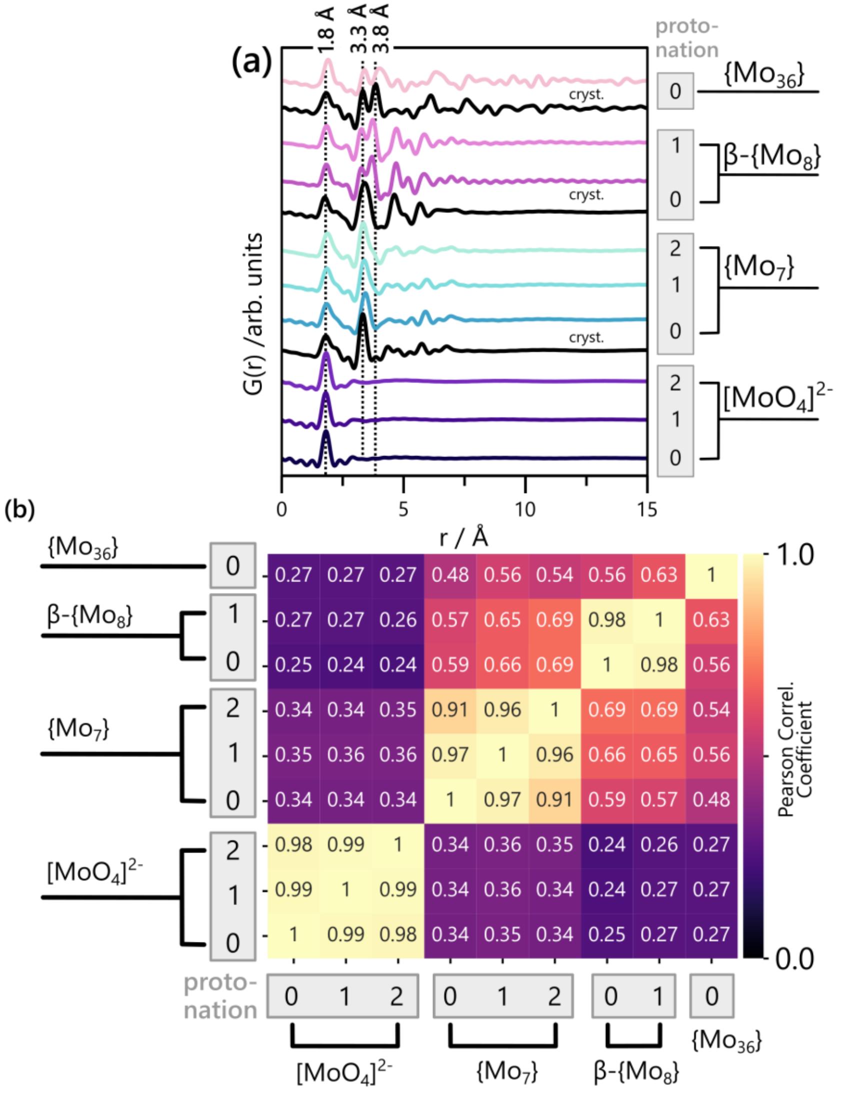

Figure S16. (a) Side-by-side comparison of all cluster structures included in the speciation models shown in Figure 4 based on their PDFs. This plot is analogous to Figure 6(a), covering a wider r-range. The same color code as in Figure 4 is used to differentiate the DFT-derived molecular geometries. PDFs of solid-state references for {Mo_7}, β-{Mo_8}, and {Mo_{36}} (indicated as "cryst.") are added in black, facilitating direct comparison with the respective DFT-derived structures. Dotted vertical lines highlight the characteristic distances of Mo-O (1.8 Å) and Mo-Mo (3.3 Å and 3.8 Å) pairs. (b) Pearson Correlation matrix for the reference PDFs, also shown in Figure 5(c), shown here including the actual Pearson Correlation coefficients for each frame combination. The color code uses dark purple and yellow to depict low and high values of the Pearson Correlation coefficient, respectively. A coefficient of 1 represents two identical PDF patterns and a coefficient of 0 represents the complete lack of similarity between two patterns.

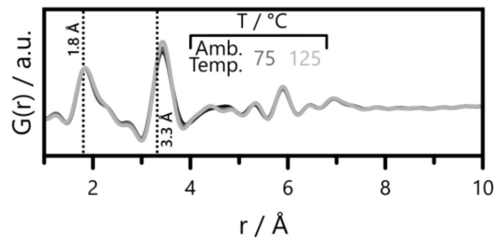

Figure S17. Comparison of the predicted PDFs for the three experimentally probed data points at pH = 3.4. The three shown PDFs equal to the $G(r)_{calc}$ patterns given in Figure 8(a). Ambient temperature, 75 °C, and 125 °C are shown in black, dark gray and light gray, respectively. The highlighted distances of 1.8 Å and 3.3 Å facilitate comparison with Figure 8(a).

References

(1) Cruywagen, J. J.; Esterhuysen, M. W.; Heyns, J. B. B. The Isolation and X-Ray Characterization of a New Sodium Heptamolybdate Compound, $Na_7[Mo_7O_{24}]OH{\cdot}21H_2O$. *Inorganica Chim. Acta* **2003**, *348*, 205–211. https://doi.org/10.1016/S0020-1693(02)01458-5.

(2) Hakouk, K.; Oms, O.; Dolbecq, A.; El Moll, H.; Marrot, J.; Evain, M.; Molton, F.; Duboc, C.; Deniard, P.; Jobic, S.; Mialane, P.; Dessapt, R. Sulfonium Polyoxometalates: A New Class of Solid-State Photochromic Hybrid Organic–Inorganic Materials. *Inorg. Chem.* **2013**, *52* (2), 555–557. https://doi.org/10.1021/ic302477p.

(3) Krebs, B.; Stiller, S.; Tytko, K. H.; Mehmke, J. Structure and Bonding in the High Molecular Weight Isopolymolybdate Ion, $[Mo_{36}O_{112}(H_2O)_{16}]^{8-}$ The Crystal Structure of $Na_8[Mo_{36}O_{112}(H_2O)_{16}]$ 58 H_2O. *Eur. J. Solid State Inorg. Chem.* **1991**, *28* (5), 883–903.

(4) Cruywagen, J. J. Protonation, Oligomerization, and Condensation Reactions of Vanadate(V), Molybdate(VI), and Tungstate(VI). In *Advances in Inorganic Chemistry*; Sykes, A. G., Ed.; Academic Press, 1999; Vol. 49, pp 127–182. https://doi.org/10.1016/S0898-8838(08)60270-6.

(5) Wang, S.-Y.; Dong, X.; Zhou, Z.-H. Novel Isopolymolybdates with Different Configurations of Hexagram, Double Dish, and Triangular Dodecahedron. *J. Solid State Chem.* **2021**, *300*, 122229. https://doi.org/10.1016/j.jssc.2021.122229.

(6) Lemmon, Eric W.; Bell, Ian H.; Huber, Marcia L.; McLinden, Mark O. *"Thermophysical Properties of Fluid Systems" in NIST Chemistry WebBook, NIST Standard Reference Database Number 69*; Eds. P. J. Linstrom and W. G. Mallard, National Institute of Standards and Technology: Gaithersburg MD, 20899. https://doi.org/10.18434/T4D303, (retrieved November 25, 2024).

(7) Klamt, A.; Schüürmann, G. COSMO: A New Approach to Dielectric Screening in Solvents with Explicit Expressions for the Screening Energy and Its Gradient. *J. Chem. Soc. Perkin Trans. 2* **1993**, No. 5, 799–805. https://doi.org/10.1039/P29930000799.

(8) Petrus, E.; Buils, J.; Garay-Ruiz, D.; Segado-Centellas, M.; Bo, C. POMSimulator: An Open-Source Tool for Predicting the Aqueous Speciation and Self–Assembly Mechanisms of Polyoxometalates. *J. Comput. Chem.* **2024**, *45*, 2242–2250. https://doi.org/10.1002/jcc.27389.
